# Long-term inpatient disease burden in the Adult Life after Childhood Cancer in Scandinavia (ALiCCS) study: A cohort study of 21,297 childhood cancer survivors

**DOI:** 10.1371/journal.pmed.1002296

**Published:** 2017-05-09

**Authors:** Sofie de Fine Licht, Kathrine Rugbjerg, Thorgerdur Gudmundsdottir, Trine G. Bonnesen, Peter Haubjerg Asdahl, Anna Sällfors Holmqvist, Laura Madanat-Harjuoja, Laufey Tryggvadottir, Finn Wesenberg, Henrik Hasle, Jeanette F. Winther, Jørgen H. Olsen, on behalf of the ALiCCS study group

**Affiliations:** 1 Danish Cancer Society Research Center, Copenhagen, Denmark; 2 Department of Paediatrics, Aarhus University Hospital, Aarhus, Denmark; 3 Paediatric Oncology and Haematology, Skane University Hospital, Lund, Sweden; 4 Department of Clinical Sciences, Lund University, Lund, Sweden; 5 Finnish Cancer Registry, Helsinki, Finland; 6 Department of Paediatrics, University of Helsinki, Finland; 7 Helsinki University Hospital, Helsinki, Finland; 8 Icelandic Cancer Registry, Reykjavik, Iceland; 9 Faculty of Medicine, University of Iceland, Reykjavik, Iceland; 10 Cancer Registry of Norway, Oslo, Norway; 11 Department of Paediatric Medicine, Oslo University Hospital, Oslo, Norway; 12 Institute of Clinical Medicine, Faculty of Medicine, University of Oslo, Oslo, Norway; Stanford University, UNITED STATES

## Abstract

**Background:**

Survivors of childhood cancer are at increased risk for a wide range of late effects. However, no large population-based studies have included the whole range of somatic diagnoses including subgroup diagnoses and all main types of childhood cancers. Therefore, we aimed to provide the most detailed overview of the long-term risk of hospitalisation in survivors of childhood cancer.

**Methods and findings:**

From the national cancer registers of Denmark, Finland, Iceland, and Sweden, we identified 21,297 5-year survivors of childhood cancer diagnosed with cancer before the age of 20 years in the periods 1943–2008 in Denmark, 1971–2008 in Finland, 1955–2008 in Iceland, and 1958–2008 in Sweden. We randomly selected 152,231 population comparison individuals matched by age, sex, year, and country (or municipality in Sweden) from the national population registers. Using a cohort design, study participants were followed in the national hospital registers in Denmark, 1977–2010; Finland, 1975–2012; Iceland, 1999–2008; and Sweden, 1968–2009. Disease-specific hospitalisation rates in survivors and comparison individuals were used to calculate survivors’ standardised hospitalisation rate ratios (RRs), absolute excess risks (AERs), and standardised bed day ratios (SBDRs) based on length of stay in hospital. We adjusted for sex, age, and year by indirect standardisation. During 336,554 person-years of follow-up (mean: 16 years; range: 0–42 years), childhood cancer survivors experienced 21,325 first hospitalisations for diseases in one or more of 120 disease categories (cancer recurrence not included), when 10,999 were expected, yielding an overall RR of 1.94 (95% confidence interval [95% CI] 1.91–1.97). The AER was 3,068 (2,980–3,156) per 100,000 person-years, meaning that for each additional year of follow-up, an average of 3 of 100 survivors were hospitalised for a new excess disease beyond the background rates. Approximately 50% of the excess hospitalisations were for diseases of the nervous system (19.1% of all excess hospitalisations), endocrine system (11.1%), digestive organs (10.5%), and respiratory system (10.0%). Survivors of all types of childhood cancer were at increased, persistent risk for subsequent hospitalisation, the highest risks being those of survivors of neuroblastoma (RR: 2.6 [2.4–2.8]; *n* = 876), hepatic tumours (RR: 2.5 [2.0–3.1]; *n* = 92), central nervous system tumours (RR: 2.4 [2.3–2.5]; *n* = 6,175), and Hodgkin lymphoma (RR: 2.4 [2.3–2.5]; *n* = 2,027). Survivors spent on average five times as many days in hospital as comparison individuals (SBDR: 4.96 [4.94–4.98]; *n* = 422,218). The analyses of bed days in hospital included new primary cancers and recurrences. Of the total 422,218 days survivors spent in hospital, 47% (197,596 bed days) were for new primary cancers and recurrences. Our study is likely to underestimate the absolute overall disease burden experienced by survivors, as less severe late effects are missed if they are treated sufficiently in the outpatient setting or in the primary health care system.

**Conclusions:**

Childhood cancer survivors were at increased long-term risk for diseases requiring inpatient treatment even decades after their initial cancer. Health care providers who do not work in the area of late effects, especially those in primary health care, should be aware of this highly challenged group of patients in order to avoid or postpone hospitalisations by prevention, early detection, and appropriate treatments.

## Abstract 2

In this population-based cohort study in Denmark, Finland, Iceland, and Sweden, Sofie de Fine Licht and colleagues present the increased long-term burden of disease serious enough to require hospitalization in survivors of childhood cancer.

## Author summary

**Why was this study done?:**

- Today, four out of five children with cancer become long-term survivors. However, as toxic treatments, often in combination, are given at an age distinguished by growth and organ maturation, many childhood cancer survivors face significant and often uncharacterised late somatic morbidities.
- No previous study has been able to examine the long-term inpatient morbidity in childhood cancer survivors including virtually all combinations of type of childhood cancer and disease-specific outcome.

**What did the researchers do and find?:**

- We explored the lifetime somatic morbidity pattern in 21,297 5-year survivors of childhood cancer using the unique health registries of the Nordic countries, including 120 disease entities and all main diagnostic groups of childhood cancer.
- We found that survivors had an overall 2-fold risk of being hospitalised and experienced longer stays in hospital than population comparisons of similar age and sex.
- Major reasons for hospitalisation among cancer survivors were diseases of the nervous system (19.1% of all excess hospitalisations), endocrine system (11.1%), digestive organs (10.5%), and respiratory system (10.0%). Together, these four groups of diseases accounted for 51% of the excess cause-specific hospitalisations among cancer survivors.
- The morbidity pattern was highly dependent on the type of childhood cancer, with highest risks seen for survivors of neuroblastoma, hepatic and central nervous system (CNS) tumours, and Hodgkin lymphoma.

**What do these findings mean?:**

- This study provides a comprehensive overview of the lifelong, complex, and often serious disease pattern that childhood cancer patients encounter after ended treatment.
- As we have used diagnostic inpatient information as measure of outcomes, our study presents the most serious somatic disease burden, which, in combination with previously described cognitive and other psychological adverse effects, may have significant impact on the quality of life and overall mortality of childhood cancer survivors.
- Clinicians in primary and secondary health care should be aware of this vulnerable group of patients for better detection, prevention, and management of treatment-induced late effects.
- We recommend long-term follow-up of high-risk subsets of childhood cancer survivors in a specialised setting.

## Introduction

The number of childhood cancer survivors is increasing steadily in many parts of the world because of the extraordinary improvement in survival rates during the past five decades [1]. In the Nordic countries, four of five childhood cancer patients can expect to be long-term survivors [2]. These major improvements in survival come, however, at a price. Because of intense exposure to radiation and highly toxic compounds during treatment, a high proportion of survivors of childhood cancer now face somatic, mental, and cognitive late effects, many of which become clinically apparent even decades after the cancer was cured [3–7]. Only a limited number of studies have investigated the hospitalisation pattern subsequent to treatment for cancer diagnosed during childhood, adolescence, and young adulthood [8–13]. They all revealed an overall increased risk for hospitalisation. However, the record linkage studies with diagnostic inpatient information [9,10,12,13] were limited by study size and unable to give reliable risk estimates for rare types of childhood cancer or combinations of types of childhood cancer and types of subsequent disease. The larger studies were either based on self-reported information on causes for hospitalisation [7] or questionnaire data from patients’ primary health care physicians [10], with a relatively large proportion of survivors lost to follow-up.

In a population-based cohort study with virtually no loss to follow-up and exclusive use of medically verified diagnostic information from individual inpatient records, we studied the full range of somatic morbid conditions requiring hospitalisation in 21,297 5-year survivors of childhood cancer diagnosed between 1943–2008. As this is the largest long-term follow-up study of inpatient care among childhood cancer survivors conducted so far, it allowed stratifications and detailed analyses that were not possible in previous studies and provides novel information on diseases that first become symptomatic in middle age or senescence. Thus, the primary aim of our study was to present a comprehensive yet detailed overview of the long-term frequency and distribution of somatic diseases serious enough to require hospitalisations in survivors of childhood cancer combined and by type of cancer.

## Methods

### Cancer survivor and comparison cohorts

This retrospective, register-based cohort study is part of the collaborative study Adult Life after Childhood Cancer in Scandinavia (ALiCCS) (www.aliccs.org) [14]. The ALiCCS study was approved by the national bioethics committee, the data protection authority, or the national institute for health and welfare in the respective countries (Denmark: 2010-41-4334; Finland: THL/520/5.05.00/2016; Iceland: VSN 10–041; and Sweden: Ö 10–2010, 2011/19). Consent from study participants was not required as all data were available in national health registers.

**Fig 1:**
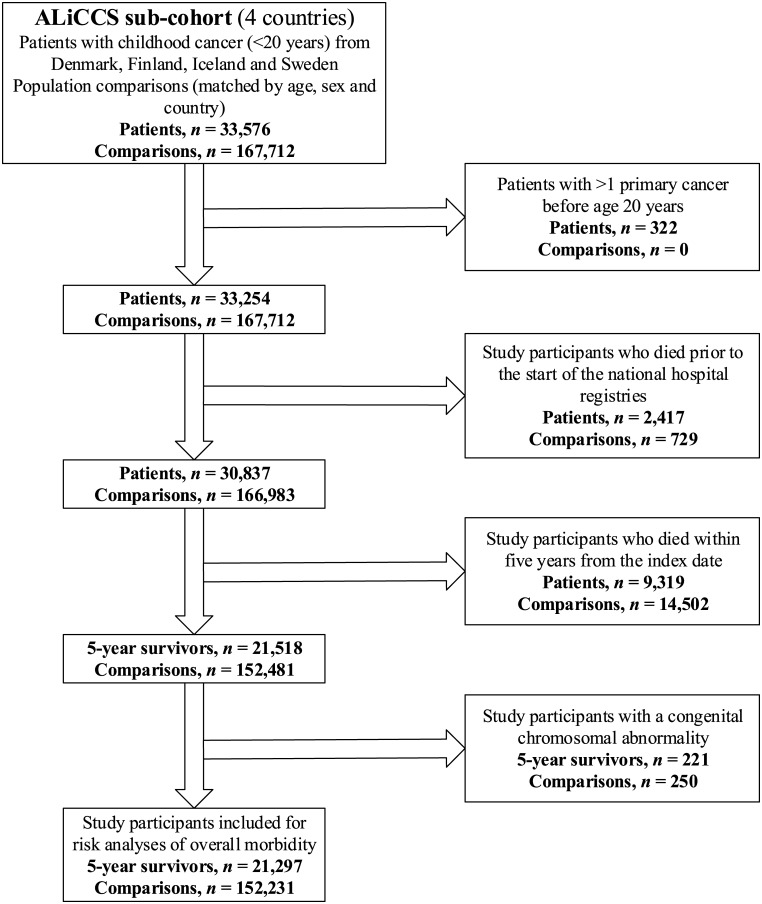
Flow diagram of exclusions of study participants and final study cohort size. Note: the index date is the date of cancer diagnosis and the corresponding date for population comparisons.

The basic childhood cancer cohort in the present analysis is a subcohort of the Nordic ALiCCS material, comprising 33,576 individuals with cancer diagnosed in Denmark, Finland, Iceland, or Sweden in people under the age of 20 years in the periods 1943–2008 in Denmark, 1971–2008 in Finland, 1955–2008 in Iceland, and 1958–2008 in Sweden (Fig 1) [15–18]. Patients from Norway were not included, as complete hospitalisation histories with all diseases included in the present study were not available. From the cancer registries, we obtained each patient’s personal identification number, date of diagnosis, and type of cancer and assigned patients to the 12 main diagnostic groups of the International Classification Scheme for Childhood Cancer, with lymphoma divided further into Hodgkin, non-Hodgkin lymphoma, and other lymphomas [19]. For each childhood cancer patient, we randomly selected five comparison individuals from the national population registers who were alive on the date of the cancer diagnosis of the corresponding patient; of the same sex, age, and country; and without a cancer diagnosis before the age of 20 years. Fewer than five comparison individuals were available for 157 patients, leaving 167,712 participants for study. For both patients and comparison individuals, we obtained information from the population registers on vital status and emigration during follow-up.

Before linkage of study participants to the respective national hospital registers, we excluded those in whom more than one cancer was diagnosed during childhood, those who had died or emigrated before the start of the national hospital registers (Sweden, stepwise inclusion of counties in 1964–1987 and nationwide since 1987; Finland, 1975; Denmark, 1977; Iceland, 1999), and those who had died or emigrated within 5 years of the date of cancer diagnosis or an equivalent time lag for the population comparisons. These exclusions resulted in cohorts of 21,518 5-year childhood cancer survivors and 152,481 population comparison individuals (Fig 1).

### Hospital admissions

The nationwide hospital registries hold information on all nonpsychiatric hospital admissions in the four countries [20,21]. Registration is mandatory, and the treating physician submits diagnostic information electronically. Each admission to a hospital initiates a record, which includes the personal identification number of the patient, the dates of admission and discharge, a primary discharge diagnosis, and supplementary diagnoses coded according to the International Classification of Diseases 7th–10th revisions (ICD-7–ICD-10).

Data on cancer survivors and comparison individuals were linked to the hospital registers, and a full hospital history with discharge diagnoses was established for each person with a previous hospital contact. We excluded cancer survivors and comparison individuals who had ever been hospitalised with a congenital malformation or chromosome abnormality (ICD-8 codes 740–759, ICD-10 codes Q00–Q99), as this could possibly confound the associations between childhood cancer and several of the outcomes, leaving 21,297 5-year survivors of childhood cancer and 152,231 population comparison individuals for the risk analysis (Fig 1).

To quantify the inpatient disease burden among study participants comprehensibly, we grouped the hospital discharge diagnoses into 120 disease categories or diagnoses, which in turn were assembled into 12 main diagnostic groups (S1 Table). The 12 main diagnostic groups were mutually exclusive; all neoplasms were grouped in the two main diagnostic groups of malignant neoplasms and benign neoplasms. Diagnoses coded according to ICD-7, ICD-9, and ICD-10 were adapted to ICD-8 to the extent possible, as shown in the table. We did not include the ICD sections of ill-defined diseases and the group of injuries and violence in the analysis, as these were regarded as too unspecific for solid conclusions. Mental disorders were the focus of a previous study [3], and childbirth and pregnancy complications require special considerations and will be investigated in separate publications.

### Statistical analysis

Follow-up for diseases other than cancer was started 5 years after the date of cancer diagnosis for the cancer survivors and the corresponding date for the comparison individuals or at the beginning of the hospital registers (Denmark, 1977; Finland, 1975; Iceland, 1999; Sweden, from 1968–1987 stepwise inclusion of counties and nationwide since 1987), whichever occurred later. Follow-up for a second cancer in survivors and a first cancer in comparisons started at age 20 years at the earliest. Follow-up ended on the date of death, the date of emigration, or the end of the study (Iceland: 31 December 2008; Sweden: 31 December 2009; Denmark: 31 October 2010; Finland: 31 December 2012), whichever occurred first. As hospital registers do not reliably distinguish hospitalisations due to a relapse from those due to a primary cancer, we used the cancer registries for information on second primary cancers among childhood cancer survivors and first primary cancers among comparison individuals in analyses of hospitalisation risk. Thus, hospitalisations with childhood cancer recurrence were not included in the main analyses of hospitalisation risk.

Only the primary diagnosis, i.e., the main reason for hospitalisation at each inpatient contact, was included in the analyses. If participants had more than one hospital admission for a particular disease category, only the first record was retained. Risk was analysed for each of the 120 disease categories, and the numbers of first hospitalisations for somatic diseases in different categories were added up for the 12 main diagnostic groups. For each person, the final sum yielded the total number of first hospitalisations for diseases requiring hospitalisation in different categories. The observed number of first hospital admissions of survivors of childhood cancer for a given disease category was compared with expected numbers derived from the appropriate sex-, age-, and calendar period-specific hospitalisation rates of the comparison cohort, and the standardised hospitalisation rate ratios (RRs) were estimated. The significance and 95% confidence intervals (95% CIs) were computed using Fieller’s theorem and assuming that the observed number of first hospital contacts follows a Poisson distribution [22]. The absolute excess risk (AER), i.e., the additional risk for hospitalisation above background levels, was derived as the difference between the observed and expected first hospitalisation rates for a particular disease category per 100,000 person-years of follow-up. We did not stratify analyses by ethnicity, because this variable is not available in the Nordic population registers. However, the group of non-white individuals is historically of limited size in the Nordic countries and especially so among children.

Using the same methods as for the RR, we also added up the total number of bed days spent in hospital for cancer survivors and the number expected had they had the sex-, age-, and calendar period-specific bed day rates of the comparison individuals. We thus derived standardised bed day ratios (SBDRs) for cancer survivors. In the analyses of bed days, we included not only the first hospitalisation for a given disease category but all hospitalisations for diseases of the ICD sections of interest. Bed days for cancer recurrences were included in the analyses of SBDR.

## Results

Table 1 gives the main characteristics of the 21,297 5-year childhood cancer survivors included in the analysis. The survivors were monitored in the national hospital registers for 336,554 person-years (mean: 16 years; range: 0–42 years). Of the survivors, 27% (5,655/21,297) and 12% (2,546/21,297) were followed beyond the ages of 40 and 50 years, respectively.

**Table 1:** Characteristics of the study population of 21,297 5-year childhood cancer survivors in four Nordic countries.

Characteristic	*n*	%
**All 5-year survivors**	**21,297**	**100.0**
**Sex**		
Male	11,255	52.8
Female	10,042	47.2
**Country**		
Denmark	5,879	27.6
Finland	5,304	24.9
Iceland	333	1.6
Sweden	9,781	45.9
**Age at cancer diagnosis (years)**		
<5	6,440	30.2
5–9	3,741	17.6
10–14	4,218	19.8
15–19	6,898	32.4
**Period of cancer diagnosis**		
1943–1959	703	3.3
1960–1974	3,068	14.4
1975–1989	7,063	33.2
1990–2008	10,463	49.1
**Age at end of follow-up (years)**		
5–9	627	2.9
10–19	3,665	17.2
20–29	6,147	28.9
30–39	5,203	24.4
40–49	3,109	14.6
50–59	1,629	7.6
≥60	917	4.3
**Cancer type**		
Leukaemias	4,162	19.5
Hodgkin lymphoma	1,773	8.3
Non-Hodgkin lymphoma	1,193	5.6
Other lymphomas	205	1.0
Central nervous system tumours	4,973	23.4
Neuroblastoma	813	3.8
Retinoblastoma	578	2.7
Renal tumours	969	4.5
Hepatic tumours	117	0.5
Bone tumours	818	3.8
Soft-tissue sarcomas	1,373	6.4
Germ-cell neoplasms	1,502	7.1
Carcinomas	2,567	12.1
Other and unspecified neoplasms	254	1.2
**Type of censoring**		
Death	2,103	9.9
Emigration	410	1.9
End of follow-up (alive)	18,784	88.2

Overall, 9,698 (45.5%) childhood cancer survivors were ever admitted to hospital for somatic disease, when 5,399.2 (25.4%) were expected, yielding an RR of 1.80 (1.76–1.84). The 9,698 survivors ever hospitalised experienced 21,325 first admissions to hospital for diseases in one or more of the 120 disease categories listed in S1 Table, when 10,999.0 were expected, yielding an overall RR for a new category-specific admission of 1.94 (Table 2). The underlying RRs were 1.83 (1.78–1.87) in Denmark, 1.87 (1.82–1.92) in Finland, 1.64 (1.35–1.99) in Iceland, and 2.10 (2.05–2.15) in Sweden. Based on the observed and expected hospitalisation rates of 6,336.3 and 3,268.1 per 100,000 person-years, respectively, the AER of survivors for a new category-specific admission to hospital was 3,068 per 100,000 person-years (Table 2). Thus, for each additional year of follow-up, approximately 3 of 100 survivors of childhood cancer were hospitalised for a new excess disease. Although the relative risk was significantly increased at all ages, the degree of increase diminished substantially with increasing age, i.e., from a relative risk of 3.4 in the age group 5–9 years to 1.3 in survivors aged 60 years or older. The absolute risk did not show a similar linear decline: after about 5,700 excess category-specific hospitalisations per 100,000 person-years in the age group 5–9 years, the AERs varied from 2,500 to 3,700 excess hospitalisations per 100,000 person-years for all subsequent age groups.

**Table 2:** Observed and expected numbers of hospitalisations by sex and attained age in 21,297 5-year survivors of childhood cancer in the Nordic countries.

	5-year cancer survivors (*n*)	Disease-specific hospitalisations^a^		RR (95% CI)	Hospitalisation rates^b^		AER^b^ (95% CI)
		Observed	Expected		Observed	Expected	
**Total**	21,297	21,325	10,999.0	1.94 (1.91–1.97)	6,336.3	3,268.1	3,068 (2,980–3,156)
**Sex**							
Male	11,255	10,360	5,211.0	1.99 (1.95–2.03)	5,920.7	2,978.0	2,943 (2,825–3,060)
Female	10,042	10,965	5,788.0	1.89 (1.86–1.93)	6,786.4	3,582.3	3,204 (3,073–3,335)
**Attained age (years)**							
5–9	5,258^c^	1,105	327.5	3.4 (3.1–3.6)	8,081.4	2,395.0	5,686 (5,201–6,172)
10–19	11,420	4,560	1,881.3	2.4 (2.3–2.5)	6,157.9	2,540.5	3,617 (3,434–3,801)
20–29	14,344	6,260	3,286.9	1.9 (1.9–2.0)	5,619.9	2,950.8	2,669 (2,525–2,813)
30–39	10,644	4,420	2,442.3	1.8 (1.8–1.9)	5,723.7	3,162.7	2,561 (2,387–2,735)
40–49	5,625	2,765	1,596.5	1.7 (1.7–1.8)	7,177.8	4,144.5	3,033 (2,757–3,309)
50–59	2,542	1,525	925.9	1.6 (1.6–1.7)	9,374.2	5,691.5	3,683 (3,194–4,172)
≥60	1,062	690	538.6	1.3 (1.2–1.4)	12,712.0	9,922.0	2,790 (1,783–3,797)

95% CI, 95% confidence interval; AER, absolute excess risk; RR, standardised hospitalisation rate ratio.

^a^ Hospitalisations are for a selected set of diseases, and each person may be hospitalised for more than one disease; see Methods for details.

^b^ Hospitalisation rates and AER per 100,000 person—years.

^c^ The number of individuals under follow-up at the start of each age interval. Each individual can contribute risk time in more than one age interval; thus, the sum of all age groups exceeds 21,297.

**Fig 2:**
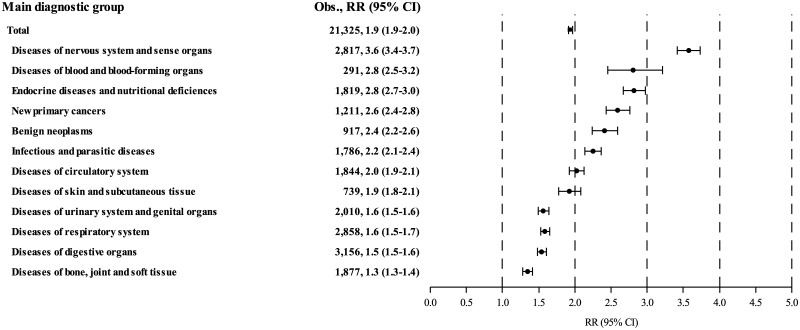
Risk of hospitalisation for somatic diseases in each of 12 main diagnostic groups. 95% CI, 95% confidence interval; Obs., observed; RR, standardised hospitalisation rate ratio.

Fig 2 shows the relative risk for hospitalisation for somatic diseases belonging to each of the 12 main diagnostic groups; the highest risks were seen for diseases of the nervous system and sense organs (RR: 3.6 [3.4–3.7]), followed by diseases of the blood and blood-forming organs (RR: 2.8 [2.5–3.2]), endocrine diseases and nutritional deficiencies (RR: 2.8 [2.7–3.0]), and new primary cancers (RR: 2.6 [2.4–2.8]).

Table 3 shows that the pattern of excess hospitalisations among survivors is dominated by diseases of the nervous system and sense organs (AER: 603 per 100,000 person-years), followed by diseases of the endocrine system and nutritional deficiencies (AER: 349 per 100,000 person-years), diseases of the digestive organs (AER: 330 per 100,000 person-years), and diseases of the respiratory system (AER: 314 per 100,000 person-years). Together, these four main diagnostic groups constituted 50.6% (1,596/3,154) of all new excess hospitalisations, mainly for epilepsy (AER: 199 per 100,000 person-years), diseases of nerves and peripheral ganglia (AER: 157 per 100,000 person-years), pneumonia (AER: 144 per 100,000 person-years), and pituitary hypofunction (AER: 101 per 100,000 person-years). Table 3 shows the estimated relative and absolute risks of cancer survivors for each of the main diagnostic groups and a selected number of disease categories. The full table including all 120 disease categories is presented in S2 Table. Particularly high relative risks were seen for pituitary hypofunction (RR: 72.0; *n* = 341), testicular dysfunction (RR: 22..9; *n* = 19), tumours of the central nervous system (CNS) (RR: 11.8; *n* = 257), and herpes zoster (RR: 11.2; *n* = 58).

**Table 3:** Standardised hospitalisation rate ratios (RRs), absolute excess risks (AERs), and the percentage (%) of total AER for main diagnostic groups and selected disease categories distinguished by AER ≥ 20.

Main diagnostic group	Number of hospitalisations^1^		RR (95% CI)	AER (95% CI)	% of total AER, (*n*/*N*)
Disease category	Observed	Expected			
**Infectious and parasitic diseases**	**1,786**	**794.6**	**2.2 (2.1**–**2.4)**	**295 (269–320)**	**9.3% (295/3,154)**
Sepsis	294	46.6	6.3 (5.4–7.3)	74 (64–84)	
Intestinal infectious diseases	515	278.0	1.9 (1.7–2.0)	72 (58–85)	
Infectious hepatitis, HIV infections (only in ICD-9 and ICD-10), and other viral diseases	294	170.4	1.7 (1.5–2.0)	37 (27–48)	
Erysipelas	180	70.5	2.6 (2.2–3.0)	33 (25–41)	
Other bacterial diseases	121	39.2	3.1 (2.5–3.8)	24 (18–31)	
**Malignant neoplasms (new primary cancer)**	**1,211**	**467.5**	**2,6 (2,4**–**2,8)**	**307 (278–336)**	**9.7% (307/3,154)**
Cancer of the eye, the brain, and other parts of central nervous system	257	23.1	11.8 (9.8–14.2)	97 (84–110)	
Cancer of the breast	182	68.2	2.7 (2.3–3.2)	47 (36–58)	
Malignant melanoma of the skin	82	36.6	2,2 (1,8–2,9)	26 (18–32)	
Cancer of the digestive organs	128	44.7	2,9 (2,3–3,5)	25 (18–32)	
**Benign neoplasms**	**917**	**380.4**	**2.4 (2.2**–**2.6)**	**159 (141–178)**	**5.1% (159/3,154)**
**Endocrine diseases, nutritional deficiencies, and other metabolic diseases**	**1,819**	**645.2**	**2.8 (2.7**–**3.0)**	**349 (323–374)**	**11.1% (349/3,154)**
Pituitary hypofunction	341	4.7	72.0 (51.8–100.1)	101 (90–112)	
Other metabolic disorders	268	30.6	8.7 (7.4–10.4)	71 (61–81)	
Disorders of other endocrine organs	251	23.2	10.8 (9.0–13.1)	68 (59–78)	
Diseases of the thyroid gland	233	77.9	3.0 (2.6–3.5)	46 (37–56)	
Abnormal menstruation	272	185.2	1.5 (1.3–1.7)	26 (16–36)	
**Diseases of the blood and blood-forming organs**	**291**	**103.6**	**2.8 (2.5**–**3.2)**	**56 (46–66)**	**1.8% (56/3,154)**
Anaemias	140	48.2	2.9 (2.4–3.5)	27 (20–34)	
**Diseases of the nervous system and sense organs**	**2,817**	**788.2**	**3.6 (3.4**–**3.7)**	**603 (571–634)**	**19.1% (603/3,154)**
Epilepsy	741	87.8	8.4 (7.6–9.3)	199 (182–215)	
Diseases of the nerves and peripheral ganglia	663	142.9	4.6 (4.2–5.1)	157 (141–172)	
Inflammatory and other diseases of the eye	492	152.3	3.2 (2.9–3.6)	103 (89–116)	
Migraine and other diseases of the brain and spinal cord	273	122.4	2.2 (2.0–2.5)	45 (35–55)	
Cataract	150	27.3	5.5 (4.5–6.8)	37 (29–44)	
Inflammatory diseases of the ear	218	115.2	1.9 (1.6–2.2)	31 (22–40)	
**Diseases of the circulatory system**	**1,844**	**912.8**	**2.0 (1.9**–**2.1)**	**277 (251–302)**	**8.8% (277/3,154)**
Cerebrovascular disease	385	127.3	3.0 (2.7–3.4)	77 (65–89)	
Heart failure	157	34.3	4.6 (3.8–5.6)	37 (29–44)	
Venous and lymphatic disease	362	267.2	1.4 (1.2–1.5)	29 (17–40)	
Ischemic heart disease	230	143.2	1.6 (1.4–1.9)	26 (17–35)	
Valvular disease (non-rheumatic)	84	15.8	5.3 (4.0–7.0)	20 (15–26)	
**Diseases of the respiratory system**	**2,858**	**1,801.6**	**1.6 (1.5**–**1.7)**	**314 (282–346)**	**10.0% (314/3,154)**
Pneumonia	737	258.8	2.8 (2.6–3.1)	144 (128–161)	
Acute upper respiratory infections	581	438.0	1.3 (1.2–1.4)	43 (28–58)	
Bronchitis and emphysema	162	81.6	2.0 (1.7–2.4)	24 (16–32)	
Respiratory failure	97	18.3	5.3 (4.1–6.8)	23 (18–29)	
**Diseases of the digestive organs**	**3,156**	**2,044.0**	**1.5 (1.5**–**1.6)**	**330 (296–364)**	**10.5% (330/3,154)**
Diseases of the teeth and supporting structures	375	140.9	2.7 (2.4–3.0)	70 (59–82)	
Paralytic ileus and intestinal obstruction	232	43.1	5.4 (4.6–6.4)	57 (48–66)	
Other diseases of the digestive system	328	168.0	2.0 (1.7–2.2)	48 (37–59)	
Diseases of the gallbladder and biliary ducts	458	312.1	1.5 (1.3–1.6)	44 (31–57)	
Diseases of the esophagus	152	57.6	2.6 (2.2–3.2)	28 (21–36)	
Diseases of the stomach and duodenum	223	131.8	1.7 (1.5–2.0)	27 (18–36)	
**Diseases of the urinary system and genital organs**	**2,010**	**1,284.8**	**1.6 (1.5**–**1.6)**	**215 (188–243)**	**6.8% (215/3,154)**
Infections of the urinary system	345	161.8	2.1 (1.9–2.4)	55 (44–66)	
Chronic cystic disease and other diseases of the breast	211	120.5	1.8 (1.5–2.0)	27 (18–36)	
Other and unspecified disorders of the urinary system	135	51.1	2.6 (2.2–3.2)	25 (18–32)	
Non-inflammatory disorders of the female genital tract	359	275.2	1.3 (1.2–1.5)	25 (14–37)	
Chronic kidney disease	104	34.0	3.1 (2.4–3.8)	21 (15–27)	
**Diseases of the skin and subcutaneous tissue**	**739**	**384.2**	**1.9 (1.8**–**2.1)**	**105 (89–122)**	**3.3% (105/3,154)**
Other disorders of the skin and subcutaneous tissue	204	42.0	4.9 (4.1–5.8)	48 (40–57)	
Infections of the skin and subcutaneous tissue	341	210.2	1.6 (1.4–1.8)	39 (28–51)	
**Diseases of the bone, joints, and soft tissue**	**1,877**	**1,392.4**	**1.3 (1.3**–**1.4)**	**144 (118–170)**	**4.6% (144/3,154)**
Osteomyelitis and other diseases of the bone and joints	985	726.3	1.4 (1.3–1.5)	79 (59–99)	
Other diseases of the musculoskeletal system	477	330.1	1.4 (1.3–1.6)	44 (31–58)	
Arthritis and rheumatism	415	329.5	1.3 (1.1–1.4)	26 (13–38)	

95% CI, 95% confidence interval; ICD, International Classification of Diseases. Note: The following chapters in the ICD-8 were not included in these analyses: 5 (“Psychiatric diseases”), 11 (“Diseases in pregnancy, during birth and perinatal diseases”), 14 (“Congenital malformations”), 15 (“Certain causes of diseases in the perinatal period and death due to this”), 16 (“Symptoms and ill-defined conditions”), 17 (“Injuries and violence”), and 18 (“External cause of accident”). Also, diseases with the following ICD-10 codes were not included in the analyses: C97, “Cancer arisen independently at several locations”; D37–D48, “Non-melanoma skin cancer”; C44, C46.0, “Neoplasms of unknown character”; and E65–E68, “Obesity” (ICD-8: 277 and ICD-9: 278).

^1^ Number of first-time hospitalisations observed and expected among survivors in defined disease categories.

**Fig 3:**
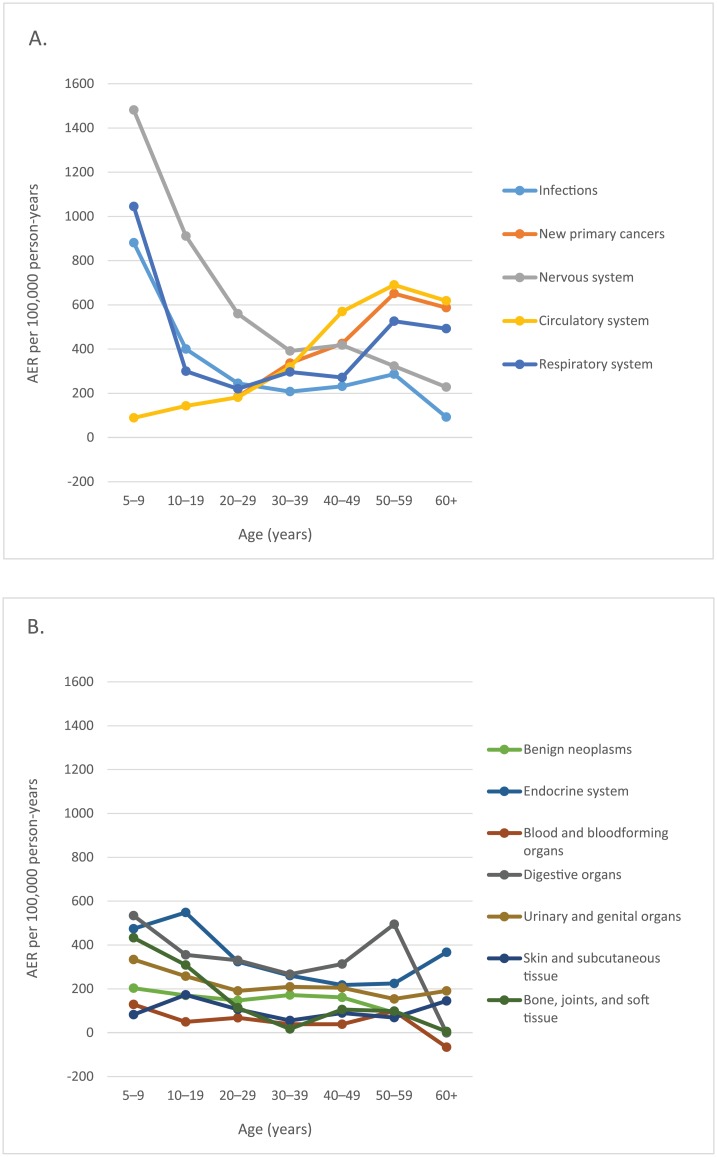
Absolute excess risks (AERs) for hospitalisation by age (years). (A) Infections, new primary cancers, and diseases of the nervous system, circulatory system, and respiratory system. Note that follow-up for new primary cancers started at age 20 years. (B) Benign neoplasms and diseases of the endocrine system; blood and blood-forming organs; digestive system; urinary and genital system; skin and subcutaneous system; and bone, joints, and soft tissue.

Fig 3A shows that the slight increase in overall AER seen in childhood cancer survivors over 40 years of age (Table 1) is due to marked increases in the AERs for diseases of the circulatory system, new primary cancers, and respiratory diseases with age. Moreover, the figure shows that the prevailing diagnostic groups during early life were diseases of the nervous system and sense organs and infectious and parasitic diseases. Except for a small peak among adolescent survivors for hospitalisation for diseases of the endocrine system, the AERs of the seven remaining main diagnostic groups did not show any appreciable variation by attained age (Fig 3B).

**Fig 4:**
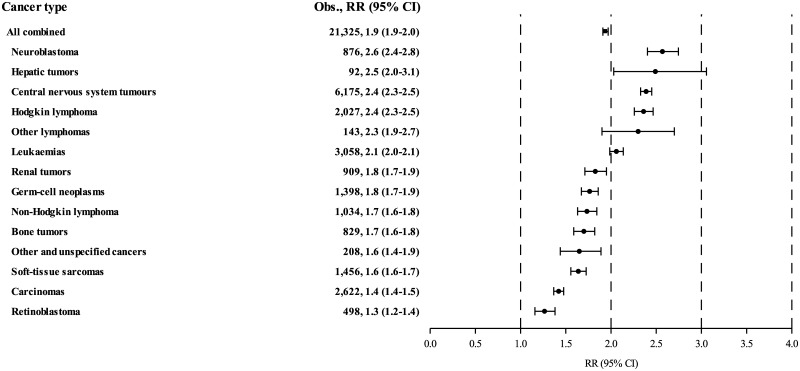
Standardised hospitalisation rate ratios (RRs) of survivors of all types of childhood cancers. 95% CI, 95% confidence interval; Obs., observed.

Survivors of all types of childhood cancers were at significantly increased risk for admission to hospital for subsequent disease (Fig 4). Survivors of neuroblastoma were at highest risk (RR: 2.6), followed by survivors of hepatic tumours (RR: 2.5), CNS tumours (RR: 2.4), Hodgkin lymphoma (RR: 2.4), and other lymphomas (RR: 2.3). Panels A—M in S1 Fig show the variations in the excess hospitalisation patterns among survivors of the 11 main groups of childhood cancer and of the two subgroups of lymphoma. For example, slightly more than half of all excess hospitalisations of survivors of CNS tumours were for subsequent diseases of the nervous system and sense organs and endocrine disorders and nutritional deficiencies, while infectious and parasitic diseases and diseases of the circulatory system were the primary reasons for hospitalisation of survivors of leukaemia and Hodgkin lymphoma, respectively.

**Fig 5:**
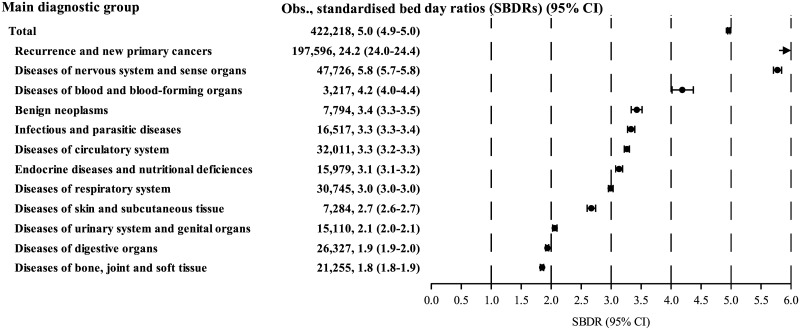
Number of bed days at hospital and associated standardised bed day ratios (SBDRs) for diseases in each of the 12 main diagnostic groups. Note that the diagnostic group “Recurrence and new primary cancers” is beyond the scale, with a standardised hospitalisation rate ratio (RR) of 24.2. 95% CI, 95% confidence interval; Obs., observed.

The 9,698 cancer survivors who were ever hospitalised spent a total of 422,218 days in hospital whereas 85,071.7 were expected, yielding an overall SBDR of 4.96 (Table 4). The highest SBDRs were seen for survivors of hepatic tumours (SBDR: 9.9), followed by survivors of leukaemia (SBDR: 8.9), neuroblastoma (SBDR: 8.4), and CNS tumours (SBDR: 6.9). Fig 5 shows that most of the days spent by cancer survivors in hospital were for cancer recurrences and new primary cancers (SBDR: 24.2), followed by diseases of the nervous system and sense organs (SBDR: 5.8), diseases of blood and blood-forming organs (SBDR: 4.2), and benign neoplasms (SBDR: 3.4). The high overall SBDR for diseases of the nervous system and sense organs was due mainly to a particularly high SBDR of 15.9 (15.5–16.2) for epilepsy. Panels A—M in S2 Fig show the SBDR distributions for survivors of each of the 11 main groups of childhood cancer and the two subgroups of lymphoma, and S3 Fig shows the SBDR distribution for cancer recurrences and new primary cancers by main group of childhood cancer.

**Table 4:** Observed and expected numbers of bed days at hospital among 21,297 5-year survivors of childhood cancer and standardised bed day ratios (SBDRs).

	Observed number of bed days	Bed day rates per 10 person-years		SBDR (95% CI)
		Observed	Expected	
**Total**	422,218	12.5	2.5	**4.96 (4.94–4.98)**
**Sex**				
Male	225,488	12.9	2.3	5.5 (5.5–5.5)
Female	196,730	12.2	2.7	4.5 (4.5–4.5)
**Age at cancer diagnosis (years)**				
0–4	107,652	10.4	1.7	6.1 (6.0–6.1)
5–9	77,998	13.4	2.1	6.5 (6.4–6.5)
10–14	86,857	13.1	2.8	4.7 (4.7–4.7)
15–19	149,711	13.8	3.4	4.1 (4.1–4.1)
**Cancer type**				
Leukaemias	76,702	14.4	1.6	8.9 (8.8–9.0)
Hodgkin lymphoma	41,568	16.3	2.7	6.2 (6.1–6.2)
Non-Hodgkin lymphoma	18,719	10.2	2.5	4.1 (4.0–4.1)
Other lymphomas	2,095	7.3	1.5	4.9 (4.7–5.1)
CNS tumours	140,967	17.7	2.5	6.9 (6.9–7.0)
Neuroblastoma	17,750	14.1	1.7	8.4 (8.2–8.5)
Retinoblastoma	5,265	4.1	2.2	1.8 (1.8–1.9)
Renal tumours	11,196	6.4	1.8	3.5 (3.5–3.6)
Hepatic tumours	2,032	14.3	1.4	9.9 (9.5–10.3)
Bone tumours	18,590	13.8	3.1	4.5 (4.4–4.5)
Soft-tissue sarcomas	25,718	10.6	3.2	3.3 (3.2–3.3)
Germ-cell neoplasms	24,435	10.1	2.7	3.7 (3.7–3.8)
Carcinomas	33,439	7.2	3.5	2.0 (2.0–2.1)
Other and unspecified neoplasms	3 742	9.4	2.6	3.6 (3.5–3.7)
**Age at hospitalisations (years)**				
5–9	30,624	22.4	1.1	20.1 (19.7–20.6)
10–19	103,919	14.0	1.4	10.0 (9.9–10.1)
20–29	132,298	11.9	1.9	6.1 (6.1–6.2)
30–39	77,628	10.1	2.5	4.1 (4.0–4.1)
40–49	42,540	11.0	3.9	2.8 (2.8–2.9)
50–59	23,858	14.7	6.5	2.2 (2.2–2.3)
≥60	11,351	20.9	12.6	1.7 (1.6–1.7)

95% CI, 95% confidence interval; CNS, central nervous system.

## Discussion

This population-based study of 21,297 5-year survivors of childhood cancer in the Nordic countries gives an extensive overview of the pattern of later somatic conditions that are serious enough to require inpatient care. The study shows that survivors are hospitalised because of a new somatic disease twice as often as population comparisons and that they spend five times as many days in hospital. Although cancer and its treatment may affect practically all organ systems adversely, the pattern of diseases requiring hospitalisation of survivors varied widely by type of childhood cancer and by survivors’ attained age. Despite the variations, however, the important findings are that the majority of childhood cancer survivors are at substantial risk for late effects requiring inpatient care and that the risk remains increased throughout life.

Our results concur with the mounting evidence of an excess risk for serious long-term morbidity in survivors of childhood cancer. Thus, in a clinical assessment, Geenen et al. identified chronic late effects in 74.5% of survivors treated in a single institution in The Netherlands [23]. Using self-reported outcomes, Oeffinger et al. showed that 62.3% of survivors included in the North American Childhood Cancer Survivor Study (CCSS) experienced at least one adverse, chronic late effect, equivalent to a 3.3-fold higher risk than their siblings [24]. This result is fairly consistent with the hospitalisation rate ratio for somatic disease of close to 2 seen in our study, taking into account differences in the sources of information and the maximum age at the end of follow-up (48 years in CCSS, lifelong in the Nordic study). In a later analysis of the CCSS material, Kurt et al. reported a survivor hospitalisation rate that was 1.6 times higher (95% CI 1.6–1.7) than that derived from a United States National Hospital Discharge Survey covering the period 1992–2005 [8].

In a few newer studies of the risk for late morbidity among survivors of cancer diagnosed during childhood, adolescence, and young adulthood (age range: 0–24 years), diagnostic information from hospital discharge registers was used as the outcome measure [9,10,12,13]. These studies, conducted in Utah, US; British Columbia, Canada; Scotland; and The Netherlands, included 1,499, 1,374, 5,229, and 1,382 survivors, respectively. They show a similar picture of substantial excess hospitalisation for a wide range of diseases, with increased cumulative numbers of days at hospital, as compared with population comparisons. Because of the substantially larger sample included in the present study and a follow-up period extending into middle age and beyond, we are able to provide a more detailed description of subsequent disease for a broader age range while maintaining relatively narrow 95% confidence limits. One notable difference between the four studies mentioned above and ours is that they included hospitalisations for psychiatric diseases, cancer recurrence, congenital abnormalities, injuries, suicide, and externally caused morbidities in the analysis, and the studies in British Columbia, Utah, and The Netherlands also included hospitalisations for pregnancy complications and delivery [9,12,13]. This probably explains the moderately higher overall risks for hospitalisation reported in those studies.

We used hospital-based diagnoses made by physicians as markers of disease outcome. Although this approach increased the validity of the diagnostic information, less severe late effects might have been missed because they were treated sufficiently as hospital outpatients or in the primary health care system. This implies that we almost certainly underestimate the absolute overall somatic disease burden experienced by childhood cancer survivors. As this limitation also applies to the comparison cohort, however, the validity of the relative risk estimates is acceptable, although restricted to conditions that require a hospital contact. Moreover, we cannot exclude the possibility that our results were affected by better medical surveillance of the survivors than the population comparisons, which could explain part of the longer hospital stays in the survivors. Not covered by the present study of the somatic disease burden but important to stress is the fact that many childhood cancer survivors face additional and sometimes significant challenges due to cognitive and other psychological adverse effects from cancer and its treatment [3,7].

The information on treatment currently included in the Nordic cancer registries is generally too crude or absent for meaningful analyses of type and dose of chemotherapy and radiation and specific disease outcomes. Although our study does not attribute causality, this comprehensive overview provides important clinical information on the lifelong inpatient disease burden experienced by childhood cancer survivors overall and of a number of patient characteristics, including type of childhood cancer, type of late effect, sex, and attained age. Associations, including dose-response effects, between specific treatment regimens and the risk of selected late effects are being addressed in clinical case-cohort studies within the ALiCCS cohort [14].

As our study is population based and includes a randomly selected comparison group and data from high-quality health registers, we consider our results valid for children treated for cancer in other countries with similar health care systems.

In conclusion, we found that survivors of childhood cancer have a highly increased long-term disease burden, with a broad range of late effects that require inpatient treatment and substantially longer stays in hospital as compared with the background population of similar age and sex. This will inevitably constitute a growing health care challenge for our society, affecting medical costs, and may profoundly influence the quality of life and life expectancy of childhood cancer survivors. Our findings underscore the need for continued follow-up of survivors, with particular focus on survivors of neuroblastoma, hepatic tumours, CNS tumours, Hodgkin lymphoma, and leukaemia. In particular, primary health care physicians should be aware of the risk for second primary cancers in patients who are childhood cancer survivors, as the relative risks for cancers are high and tumours may appear earlier in life than usual.

## Supporting information

S1 Table Definition of 120 disease categories and 12 main diagnostic groups according to the disease codes of the International Classification of Diseases, 7th–10th revision (ICD-7–ICD-10). (DOCX)

S2 Table Standardised hospitalisation rate ratios (RRs) and absolute excess risks (AERs) in the main diagnostic groups and in each of 120 somatic disease categories. (DOCX)

S1 Fig Percentage distribution of absolute excess risks (AERs) of childhood cancer survivors for hospitalisation for somatic diseases in each of 12 main diagnostic groups by type of childhood cancer. (A) Leukaemia; (B) Hodgkin lymphoma; (C) non-Hodgkin lymphoma; (D) central nervous system (CNS) tumours; (E) neuroblastoma; (F) retinoblastoma; (G) renal tumours; (H) hepatic tumours; (I) bone tumours; (J) soft-tissue sarcoma; (K) germ-cell tumours, (L) carcinomas; and (M) other and unspecified tumours. Note: other lymphomas are not presented separately because of low numbers (*n* = 205)
(DOCX)

S2 Fig Observed (Obs) number of bed days at hospital and associated standardised bed day ratios (SBDRs) for diseases in any of 120 disease categories and for somatic diseases in each of 11 main diagnostic groups by type of childhood cancer. (A) Leukaemia; (B) Hodgkin lymphoma; (C) non-Hodgkin lymphoma; (D) central nervous system (CNS) tumours; (E) neuroblastoma; (F) retinoblastoma; (G) renal tumours; (H) hepatic tumours; (I) bone tumours; (J) soft-tissue sarcoma; (K) germ-cell tumours, (L) carcinomas; and (M) other and unspecified tumours. Note: other lymphomas are not presented separately, as it is a very small group (*n* = 205). SBDRs for recurrence of childhood cancer and for new primary cancers by type of childhood cancer are presented in S3 Fig.
(DOCX)

S3 Fig Standardised bed day ratio (SBDR) with cancer recurrence or new primary cancers by childhood cancer type. (DOCX)

S1 STROBE Checklist Strengthening the Reporting of Observational Studies in Epidemiology (STROBE) checklist. (DOCX)

## Abbreviations

95% CI: 95% confidence interval
AER: absolute excess risk
ALiCCS: Adult Life after Childhood Cancer in Scandinavia
CCSS: Childhood Cancer Survivor Study
CNS: central nervous system
ICD: International Classification of Diseases
RR: standardised hospitalisation rate ratio
SBDR: standardised bed day ratio
STROBE: Strengthening the Reporting of Observational Studies in Epidemiology
