# Supplementary material for: Long-term inpatient disease burden in the Adult Life after Childhood Cancer in Scandinavia (ALiCCS) study: A cohort study of 21,297 childhood cancer survivors
Source: PLoS Med. 2017 May 9;14(5):e1002296. doi: 10.1371/journal.pmed.1002296 (PMC5423554; doi:10.1371/journal.pmed.1002296)
Supplement: S2 Table — (DOCX) [file pmed.1002296.s002.docx]

**S2 Table. Standardised hospitalisation rate ratios (RRs) and absolute excess risks (AERs) in the main diagnostic groups and in each of 120 somatic disease categories**

| **Main diagnostic group** | **No. of hospitalisations^1^** | | **RR (95% CI)** | **AER (95% CI)** | **% of total AER, (n/N)** |
| --- | --- | --- | --- | --- | --- |
| **Disease category** | **Observed** | **Expected** |  |  |  |
| **Infectious and parasitic diseases** | **1786** | **794.6** | **2.2 (2.1-2.4)** | **295 (269-320)** | **9.3% (295/3154)** |
| Intestinal infectious diseases | 515 | 278.0 | 1.9 (1.7-2.0) | 72 (58-85) |  |
| Tuberculosis | 12 | 6.4 | 1.9 (1.0-3.5) | 2 (0-4) |  |
| Sepsis | 294 | 46.6 | 6.3 (5.4-7.3) | 74 (64-84) |  |
| Erysipelas | 180 | 70.5 | 2.6 (2.2-3.0) | 33 (25-41) |  |
| Other bacterial diseases | 121 | 39.2 | 3.1 (2.5-3.8) | 24 (18-31) |  |
| Enterovirus diseases of CNS | 51 | 41.0 | 1.2 (0.9-1.7) | 3 (-1-7) |  |
| Herpes zoster | 58 | 5.2 | 11.2 (7.6-16.7) | 16 (11-20) |  |
| Other viral diseases with exanthem | 96 | 31.8 | 3.0 (2.4-3.8) | 19 (13-25) |  |
| Infectious hepatitis, HIV infections (only in ICD-9 and -10), and other viral diseases | 294 | 170.4 | 1.7 (1.5-2.0) | 37 (27-48) |  |
| Syphilis and other venereal diseases | 57 | 49.7 | 1.1 (0.9-1.5) | 2 (-2-7) |  |
| Mycoses | 23 | 6.0 | 3.8 (2.3-6.3) | 5 (2-8) |  |
| Other infectious and parasitic diseases | 85 | 47.8 | 1.8 (1.4-2.2) | 11 (6-17) |  |
| **Malignant neoplasms (new primary cancer)** | **1211** | **467.5** | **2.6 (2.4-2.8)** | **307 (278-336)** | **9.7% (307/3154)** |
| Cancer of the buccal cavity and pharynx | 25 | 8.5 | 2.9 (1.9-4.6) | 7 (3-11) |  |
| Cancer of the digestive organs | 128 | 44.7 | 2.9 (2.3-3.5) | 25 (18-32) |  |
| Cancer of the respiratory system and intrathoracic organs | 66 | 27.4 | 2.4 (1.8-3.2) | 16 (9-23) |  |
| Cancer of the bones, joints and articular cartilage | 17 | 1.8 | 9.3 (4.6-18.4) | 6 (3-10) |  |
| Malignant melanoma of the skin | 82 | 36.6 | 2.2 (1.8-2.9) | 26 (18-32) |  |
| Cancer of the mesothelium and connective tissue | 52 | 6.7 | 7.8 (5.3-11.3) | 19 (13-25) |  |
| Cancer of the breast | 182 | 68.2 | 2.7 (2.3-3.2) | 47 (36-58) |  |
| Cancer of the female genital organs incl. skin | 159 | 143.9 | 1.1 (0.9-1.3) | 6 (-4-17) |  |
| Cancer of the male genital organs incl. skin | 36 | 37.0 | 1.0 (0.7-1.4) | 0 (-6-5) |  |
| Cancer of the urinary tract | 43 | 17.8 | 2.4 (1.7-3.4) | 10 (5-16) |  |
| Cancer of eye, brain and other parts of central nervous system | 257 | 23.1 | 11.8 (9.8-14.2) | 97 (84-110) |  |
| Cancer of endocrine organs | 59 | 12.1 | 4.9 (3.5-6.7) | 19 (13-26) |  |
| Malignant lymphomas | 47 | 19.6 | 2.4 (1.7-3.3) | 11 (6-17) |  |
| Multiple myeloma | 5 | 2.5 | 2.0 (0.8-5.4) | 1 (-1-3) |  |
| Leukaemia | 17 | 8.3 | 2.2 (1.3-3.7) | 4 (0-7) |  |
| Ill-defined and unspecified cancer | 36 | 8.3 | 4.4 (3.0-6.6) | 11 (7-16) |  |
| **Benign neoplasms** | **917** | **380.4** | **2.4 (2.2-2.6)** | **159 (141-178)** | **5.1% (159/3154)** |
| **Endocrine diseases, nutritional deficiencies, and other metabolic diseases** | **1819** | **645.2** | **2.8 (2.7-3.0)** | **349 (323-374)** | **11.1% (349/3154)** |
| Diseases of the thyroid gland | 233 | 77.9 | 3.0 (2.6-3.5) | 46 (37-56) |  |
| Diabetes mellitus | 200 | 143.1 | 1.4 (1.2-1.6) | 17 (8-26) |  |
| Other disorders of glucose regulation and pancreatic internal secretion | 26 | 11.6 | 2.2 (1.4-3.4) | 4 (1-7) |  |

**S2 Table continued**

| **Main diagnostic group** | **No. of hospitalisations^1^** | | **RR (95% CI)** | **AER (95% CI)** | **% of total AER, (n/N)** |
| --- | --- | --- | --- | --- | --- |
| **Disease category** | **Observed** | **Expected** |  |  |  |
| Pituitary hypofunction | 341 | 4.7 | 72.0 (51.8-100.1) | 101 (90-112) |  |
| Ovarian dysfunction | 8 | 2.9 | 2.8 (1.3-6.2) | 2 (0-3) |  |
| Testicular dysfunction | 19 | 0.8 | 22.9 (9.5-54.7) | 5 (3-8) |  |
| Disorders of other endocrine organs | 251 | 23.2 | 10.8 (9.0-13.1) | 68 (59-78) |  |
| Nutritional deficiencies | 21 | 5.8 | 3.6 (2.2-6.0) | 5 (2-7) |  |
| Other metabolic disorders | 268 | 30.6 | 8.7 (7.4-10.4) | 71 (61-81) |  |
| Male sterility | 18 | 4.7 | 3.8 (2.2-6.7) | 4 (1-6) |  |
| Abnormal menstruation | 272 | 185.2 | 1.5 (1.3-1.7) | 26 (16-36) |  |
| Female infertility | 104 | 106.5 | 1.0 (0.8-1.2) | -1 (-7-6) |  |
| Other disorders of the female reproductive system | 58 | 45.3 | 1.3 (1.0-1.7) | 4 (-1-8) |  |
| **Diseases of the blood and blood-forming organs** | **291** | **103.6** | **2.8 (2.5-3.2)** | **56 (46-66)** | **1.8% (56/3154)** |
| Anaemias | 140 | 48.2 | 2.9 (2.4-3.5) | 27 (20-34) |  |
| Coagulation defects, purpura, and other haemorrhagic conditions | 59 | 20.4 | 2.9 (2.2-3.9) | 11 (7-16) |  |
| Agranulocytosis | 24 | 4.4 | 5.5 (3.3-9.2) | 6 (3-9) |  |
| Other diseases of the blood and blood-forming organs | 68 | 30.6 | 2.2 (1.7-2.9) | 11 (6-16) |  |
| **Diseases of the nervous system and sense organs** | **2817** | **788.2** | **3.6 (3.4-3.7)** | **603 (571-634)** | **19.1% (603/3154)** |
| Meningitis | 50 | 17.8 | 2.8 (2.0-3.9) | 10 (5-14) |  |
| Other inflammatory diseases of CNS | 44 | 9.7 | 4.5 (3.1-6.6) | 10 (6-14) |  |
| Multiple sclerosis and other demyelinating diseases of CNS | 29 | 25.4 | 1.1 (0.8-1.7) | 1 (-2-4) |  |
| Parkinson disease and other movement disorders | 27 | 7.9 | 3.4 (2.2-5.3) | 6 (3-9) |  |
| Epilepsy | 741 | 87.8 | 8.4 (7.6-9.3) | 199 (182-215) |  |
| Migraine and other diseases of the brain and spinal cord | 273 | 122.4 | 2.2 (2.0-2.5) | 45 (35-55) |  |
| Senile and pre-senile dementia | 14 | 3.2 | 4.3 (2.3-8.3) | 3 (1-5) |  |
| Diseases of the nerves and peripheral ganglia | 663 | 142.9 | 4.6 (4.2-5.1) | 157 (141-172) |  |
| Inflammatory and other diseases of the eye | 492 | 152.3 | 3.2 (2.9-3.6) | 103 (89-116) |  |
| Cataract | 150 | 27.3 | 5.5 (4.5-6.8) | 37 (29-44) |  |
| Inflammatory diseases of the ear | 218 | 115.2 | 1.9 (1.6-2.2) | 31 (22-40) |  |
| Ménière's disease and otosclerosis | 47 | 41.8 | 1.1 (0.8-1.5) | 2 (-3-6) |  |
| Other diseases of the ear and deafness | 69 | 27.9 | 2.5 (1.9-3.2) | 12 (7-17) |  |
| **Diseases of the circulatory system** | **1844** | **912.8** | **2.0 (1.9-2.1)** | **277 (251-302)** | **8.8% (277/3154)** |
| Acute rheumatic fever | 1 | 2.4 | 0.4 (0.1-3.1) | 0 (-1-0) |  |
| Chronic rheumatic heart disease | 12 | 3.1 | 3.9 (2.0-7.8) | 3 (1-5) |  |
| Hypertensive disease | 121 | 55.8 | 2.2 (1.8-2.7) | 19 (13-26) |  |
| Ischemic heart disease | 230 | 143.2 | 1.6 (1.4-1.9) | 26 (17-35) |  |
| Pulmonary heart disease | 79 | 26.7 | 3.0 (2.3-3.8) | 16 (10-21) |  |
| Pericardial, myocardial, and endocardial disease | 97 | 43.0 | 2.3 (1.8-2.8) | 16 (10-22) |  |

**S2 Table continued**

| **Main diagnostic group** | **No. of hospitalisations^1^** | | **RR (95% CI)** | **AER (95% CI)** | **% of total AER, (n/N)** |
| --- | --- | --- | --- | --- | --- |
| **Disease category** | **Observed** | **Expected** |  |  |  |
| Valvular disease (non-rheumatic) | 84 | 15.8 | 5.3 (4.0-7.0) | 20 (15-26) |  |
| Heart failure | 157 | 34.3 | 4.6 (3.8-5.6) | 37 (29-44) |  |
| Conduction disorders | 198 | 144.7 | 1.4 (1.2-1.6) | 16 (7-25) |  |
| Cerebrovascular disease | 385 | 127.3 | 3.0 (2.7-3.4) | 77 (65-89) |  |
| Diseases of the arteries, arterioles, and capillaries | 88 | 35.6 | 2.5 (1.9-3.1) | 16 (10-21) |  |
| Venous and lymphatic disease | 362 | 267.2 | 1.4 (1.2-1.5) | 29 (17-40) |  |
| Other complications of the circulatory system | 30 | 9.8 | 3.1 (2.0-4.7) | 6 (3-9) |  |
| **Diseases of the respiratory system** | **2858** | **1801.6** | **1.6 (1.5-1.7)** | **314 (282-346)** | **10.0% (314/3154)** |
| Influenza | 87 | 42.1 | 2.1 (1.6-2.6) | 13 (8-19) |  |
| Acute upper respiratory infections | 581 | 438.0 | 1.3 (1.2-1.4) | 43 (28-58) |  |
| Other disorders of the upper respiratory tract | 792 | 783.7 | 1.0 (0.9-1.1) | 3 (-15-20) |  |
| Pneumonia | 737 | 258.8 | 2.8 (2.6-3.1) | 144 (128-161) |  |
| Abscess of lung and pyothorax | 76 | 22.8 | 3.3 (2.6-4.4) | 16 (11-21) |  |
| Bronchitis and emphysema | 162 | 81.6 | 2.0 (1.7-2.4) | 24 (16-32) |  |
| Asthma | 117 | 104.0 | 1.1 (0.9-1.4) | 4 (-3-11) |  |
| Lung diseases due to external agents | 40 | 8.4 | 4.8 (3.2-7.0) | 9 (6-13) |  |
| Interstitial pulmonary diseases and pulmonary oedema | 33 | 6.9 | 4.8 (3.1-7.3) | 8 (4-11) |  |
| Pneumothorax | 64 | 32.9 | 1.9 (1.5-2.6) | 9 (4-14) |  |
| Respiratory failure | 97 | 18.3 | 5.3 (4.1-6.8) | 23 (18-29) |  |
| Other diseases of the respiratory system | 72 | 13.6 | 5.3 (3.9-7.1) | 17 (12-22) |  |
| **Diseases of the digestive organs** | **3156** | **2044.0** | **1.5 (1.5-1.6)** | **330 (296-364)** | **10.5% (330/3154)** |
| Diseases of the teeth and supporting structures | 375 | 140.9 | 2.7 (2.4-3.0) | 70 (59-82) |  |
| Other diseases of the oral cavity and salivary glands | 75 | 36.0 | 2.1 (1.6-2.7) | 12 (6-17) |  |
| Diseases of the esophagus | 152 | 57.6 | 2.6 (2.2-3.2) | 28 (21-36) |  |
| Diseases of stomach and duodenum | 223 | 131.8 | 1.7 (1.5-2.0) | 27 (18-36) |  |
| Appendicitis | 561 | 546.5 | 1.0 (0.9-1.1) | 4 (-11-19) |  |
| Hernia of the abdomal cavity | 223 | 237.8 | 0.9 (0.8-1.1) | -4 (-14-5) |  |
| Non-infective enteritis and colitis | 150 | 131.6 | 1.1 (1.0-1.4) | 5 (-2-13) |  |
| Paralytic ileus and intestinal obstruction | 232 | 43.1 | 5.4 (4.6-6.4) | 57 (48-66) |  |
| Diseases of the anal and rectal regions | 132 | 109.0 | 1.2 (1.0-1.5) | 7 (0-14) |  |
| Diseases of the peritoneum | 55 | 22.4 | 2.5 (1.8-3.3) | 10 (5-14) |  |
| Other diseases of the digestive system | 328 | 168.0 | 2.0 (1.7-2.2) | 48 (37-59) |  |
| Diseases of the liver | 100 | 47.6 | 2.1 (1.7-2.6) | 16 (10-22) |  |
| Diseases of the gallbladder and biliary ducts | 458 | 312.1 | 1.5 (1.3-1.6) | 44 (31-57) |  |
| Diseases of the pancreas | 92 | 55.1 | 1.7 (1.3-2.1) | 11 (5-17) |  |
| **Diseases of the urinary system and genital organs** | **2010** | **1284.8** | **1.6 (1.5-1.6)** | **215 (188-243)** | **6.8% (215/3154)** |
| Glomerular diseases | 54 | 25.0 | 2.2 (1.6-2.9) | 9 (4-13) |  |
| Acute renal failure | 30 | 5.2 | 5.8 (3.6-9.2) | 7 (4-11) |  |
| Chronic kidney disease | 104 | 34.0 | 3.1 (2.4-3.8) | 21 (15-27) |  |
| Urolithiasis | 147 | 101.8 | 1.4 (1.2-1.7) | 14 (6-21) |  |

**S2 Table continued**

| **Main diagnostic group** | **No. of hospitalisations^1^** | | **RR (95% CI)** | **AER (95% CI)** | **% of total AER, (n/N)** |
| --- | --- | --- | --- | --- | --- |
| **Disease category** | **Observed** | **Expected** |  |  |  |
| Obstructive uropathy | 81 | 35.0 | 2.3 (1.8-3.0) | 14 (8-19) |  |
| Infections of the urinary system | 345 | 161.8 | 2.1 (1.9-2.4) | 55 (44-66) |  |
| Other and unspecified disorders of the urinary system | 135 | 51.1 | 2.6 (2.2-3.2) | 25 (18-32) |  |
| Diseases of the prostate | 27 | 23.0 | 1.2 (0.8-1.8) | 1 (-2-4) |  |
| Hydrocoele and spermatocoele | 66 | 17.3 | 3.8 (2.9-5.1) | 15 (10-19) |  |
| Orchitis and epididymis | 33 | 23.7 | 1.4 (1.0-2.0) | 3 (-1-6) |  |
| Other diseases of the male genital organs | 140 | 119.1 | 1.2 (1.0-1.4) | 6 (-1-14) |  |
| Chronic cystic disease and other diseases of the breast | 211 | 120.5 | 1.8 (1.5-2.0) | 27 (18-36) |  |
| Inflammatory diseases of the female pelvic organs | 192 | 197.3 | 1.0 (0.8-1.1) | -2 (-10-7) |  |
| Endometriosis | 86 | 92.1 | 0.9 (0.7-1.2) | -2 (-8-4) |  |
| Non-inflammatory disorders of the female genital tract | 359 | 275.2 | 1.3 (1.2-1.5) | 25 (14-37) |  |
| **Diseases of the skin and subcutaneous tissue** | **739** | **384.2** | **1.9 (1.8-2.1)** | **105 (89-122)** | **3.3% (105/3154)** |
| Infections of the skin and subcutaneous tissue | 341 | 210.2 | 1.6 (1.4-1.8) | 39 (28-51) |  |
| Other inflammatory conditions of the skin and subcutaneous tissue | 127 | 95.2 | 1.3 (1.1-1.6) | 9 (3-16) |  |
| Radiodermatitis | 1 | 0.0 | N/A | 0 (0-1) |  |
| Disorders of skin appendages (hair, nails, sweat glands) | 66 | 35.8 | 1.8 (1.4-2.4) | 9 (4-14) |  |
| Other disorders of the skin and subcutaneous tissue | 204 | 42.0 | 4.9 (4.1-5.8) | 48 (40-57) |  |
| **Diseases of the bone, joints and soft tissue** | **1877** | **1392.4** | **1.3 (1.3-1.4)** | **144 (118-170)** | **4.6% (144/3154)** |
| Arthritis and rheumatism | 415 | 329.5 | 1.3 (1.1-1.4) | 26 (13-38) |  |
| Osteomyelitis and other diseases of the bone and joints | 985 | 726.3 | 1.4 (1.3-1.5) | 79 (59-99) |  |
| Other diseases of the musculoskeletal system | 477 | 330.1 | 1.4 (1.3-1.6) | 44 (31-58) |  |
| ^1^ Number of first-time hospitalisations observed and expected among survivors in defined disease categories. **Note:** The following chapters in the ICD-8 were not included in these analyses: 5 (Psychiatric diseases), 11 (Diseases in pregnancy, during birth and perinatal diseases), 14 (Congenital malformations), 15 (Certain causes of diseases in the perinatal period and death due to this), 16 (Symptoms and ill-defined conditions), 17 (Injuries and violence), 18 (External cause of accident). Also, diseases with the following ICD-10 codes were not included in the analyses: C97, Cancer arisen independently at several locations; D37–D48, Non-melanoma skin cancer; C44, C46.0, Neoplasms of unknown character; E65–E68, Obesity (ICD-8: 277 and ICD-9: 278). | | | | | |
