# Supplementary material for: Long-term inpatient disease burden in the Adult Life after Childhood Cancer in Scandinavia (ALiCCS) study: A cohort study of 21,297 childhood cancer survivors
Source: PLoS Med. 2017 May 9;14(5):e1002296. doi: 10.1371/journal.pmed.1002296 (PMC5423554; doi:10.1371/journal.pmed.1002296)
Supplement: S1 Fig — (A) Leukaemia; (B) Hodgkin lymphoma; (C) non-Hodgkin lymphoma; (D) central nervous system (CNS) tumours; (E) neuroblastoma; (F) retinoblastoma; (G) renal tumours; (H) hepatic tumours; (I) bone tumours; (J) soft-tissue sarcoma; (K) germ-cell tumours, (L) carcinomas; and (M) other and unspecified tumours. Note: other lymphomas are not presented separately because of low numbers (n = 205) (DOCX) [file pmed.1002296.s003.docx]

**S1 Figure. Percentage distribution of absolute excess risks (AERs) of childhood cancer survivors for hospitalisation for somatic diseases in each of 12 main diagnostic groups by type of childhood cancer.**

(A) Leukaemia; (B) Hodgkin lymphoma; (C) Non-Hodgkin lymphoma; (D) CNS tumours; (E) Neuroblastoma; (F) Retinoblastoma; (G) Renal tumours; (H) Hepatic tumours; (I) Bone tumours; (J) Soft-tissue sarcoma; (K) Germ-cell tumours, (L) Carcinomas; (M) Other and unspecified tumours.

Note: Other lymphomas are not presented separately due to low numbers (n=205)

**
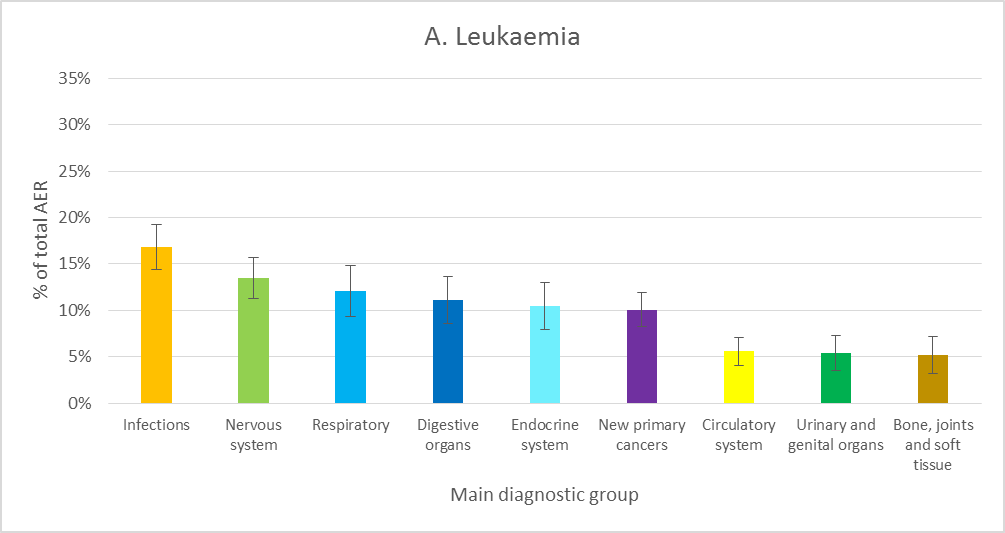
**

**Note. The following main diagnostic groups are not included: Benign neoplasms (5%), Diseases of skin and subcutaneous tissue (3%), and Diseases of blood and blood-forming organs (2%)**

**
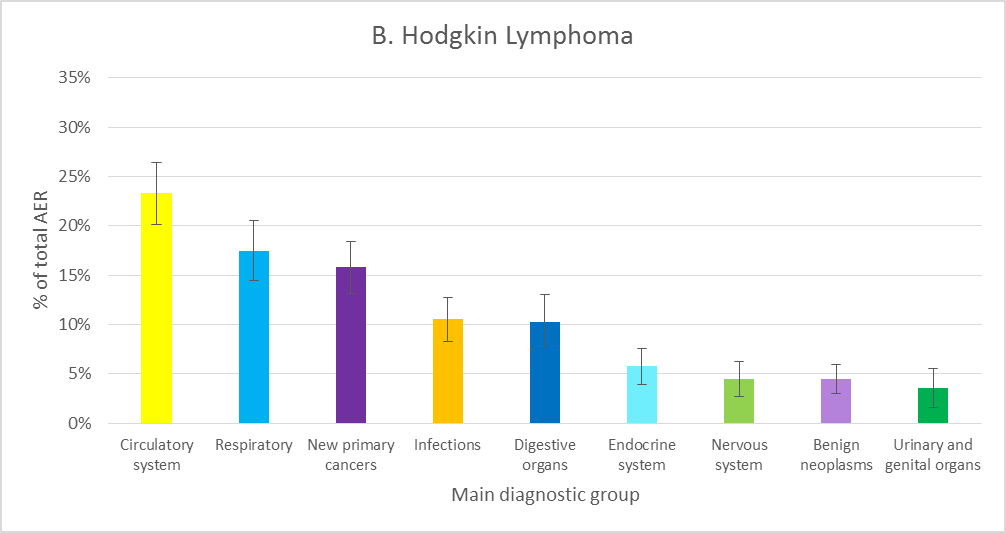
**

**Note. The following main diagnostic groups are not included: Diseases of blood and blood-forming organs (3%), Diseases of skin and subcutaneous tissue (1%), and Diseases of bone, joints and soft tissue (1%)**

**
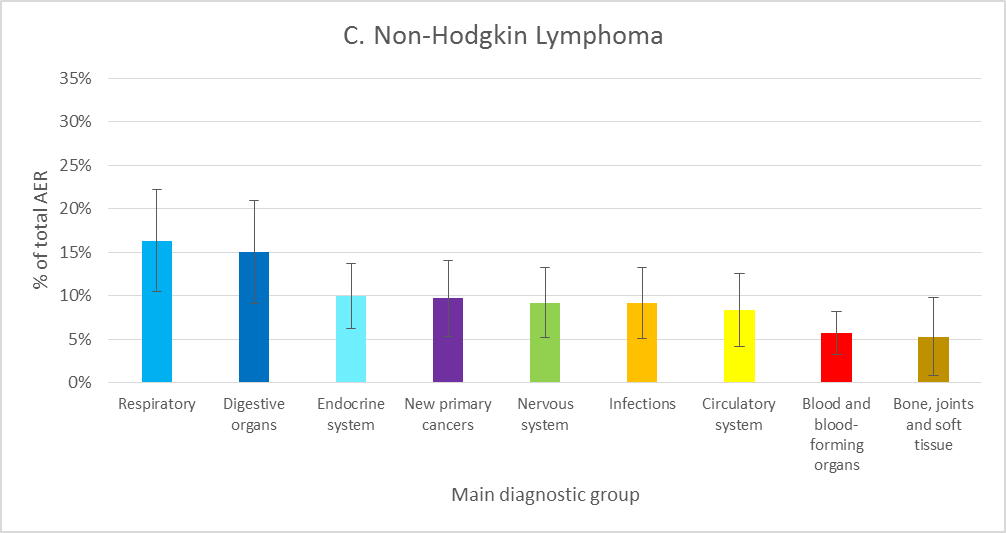
**

**Note. The following main diagnostic groups are not included: Diseases of skin and subcutaneous tissue (4%), Benign neoplasms (4%), and Diseases of urinary and genital organs (3%)**

**
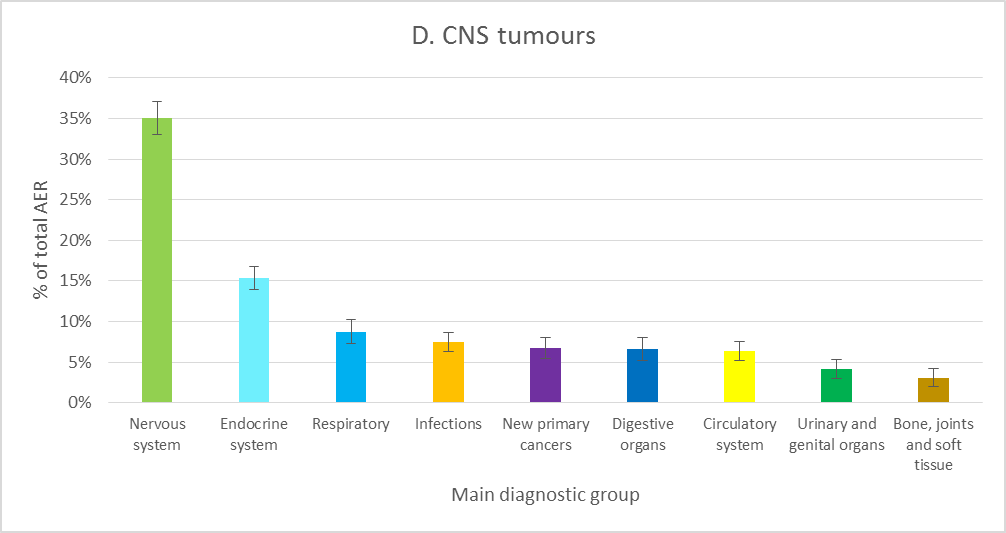
**

**Note. The scale of this figure goes up to 40%. The following main diagnostic groups are not included: Benign neoplasms (3%), Diseases of skin and subcutaneous tissue (3%), and Diseases of blood and blood-forming organs (1%)**

**
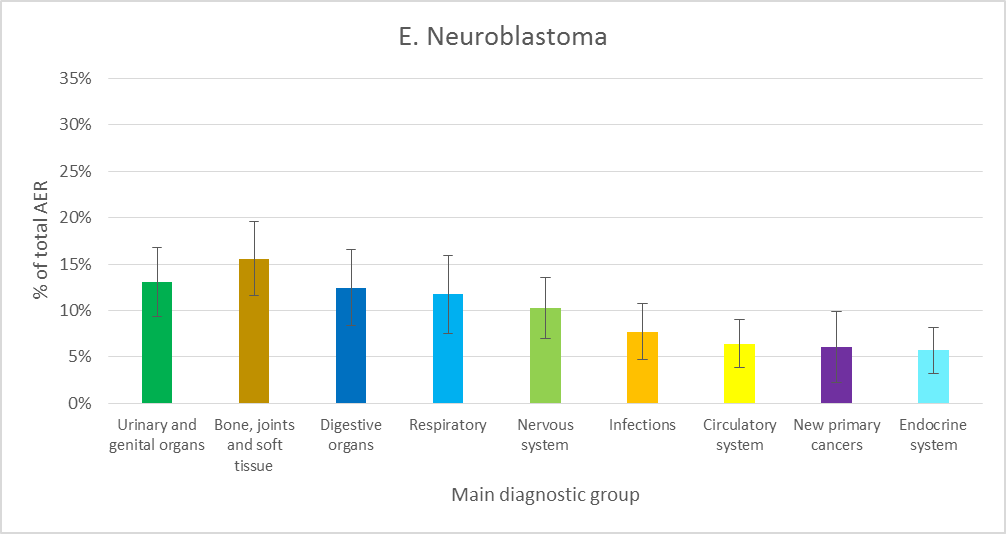
**

**Note. The following main diagnostic groups are not included: Diseases of skin and subcutaneous tissue (5%), Benign neoplasms (5%), and Diseases of blood and blood-forming organs (1%)**

**
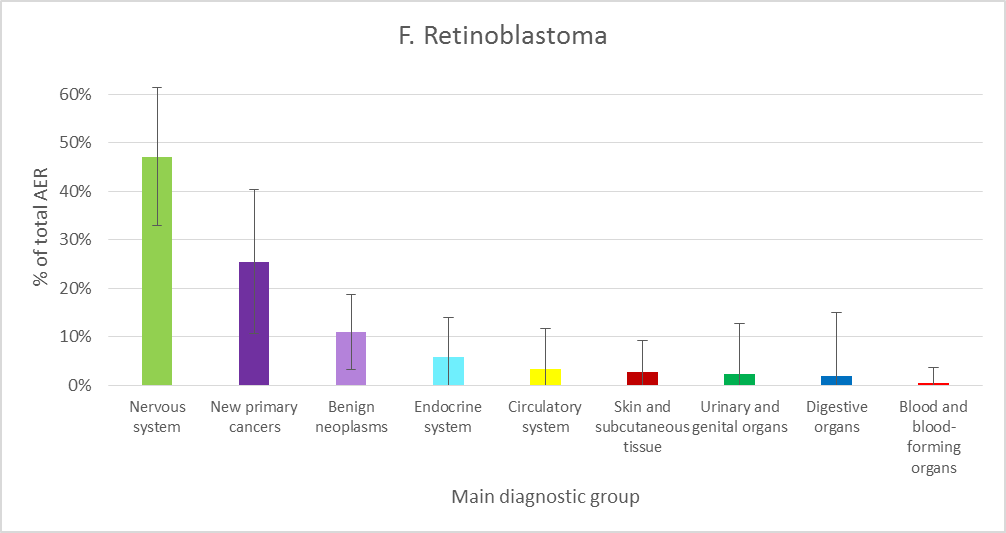
**

**Note. The scale of this figure goes up to 60%. Diseases of bone, joints and soft tissue, Infectious and parasitic diseases, and Diseases of the respiratory system are not included in this chart, as the AERs for these main diagnostic groups were below 0.**

**
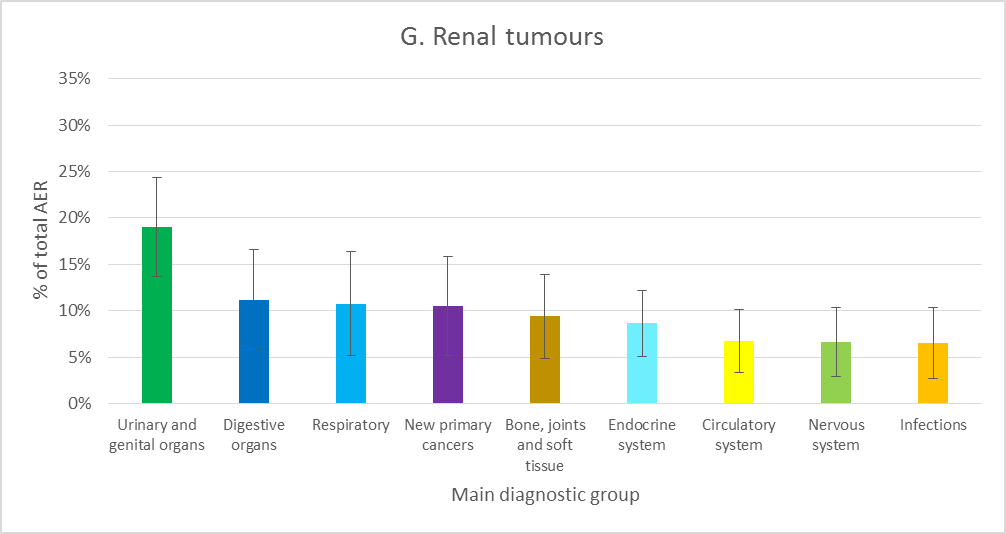
**

**Note. The following main diagnostic groups are not included: Benign neoplasms (5%), Diseases of skin and subcutaneous tissue (4%), and Diseases of blood and blood-forming organs (1%)**

**
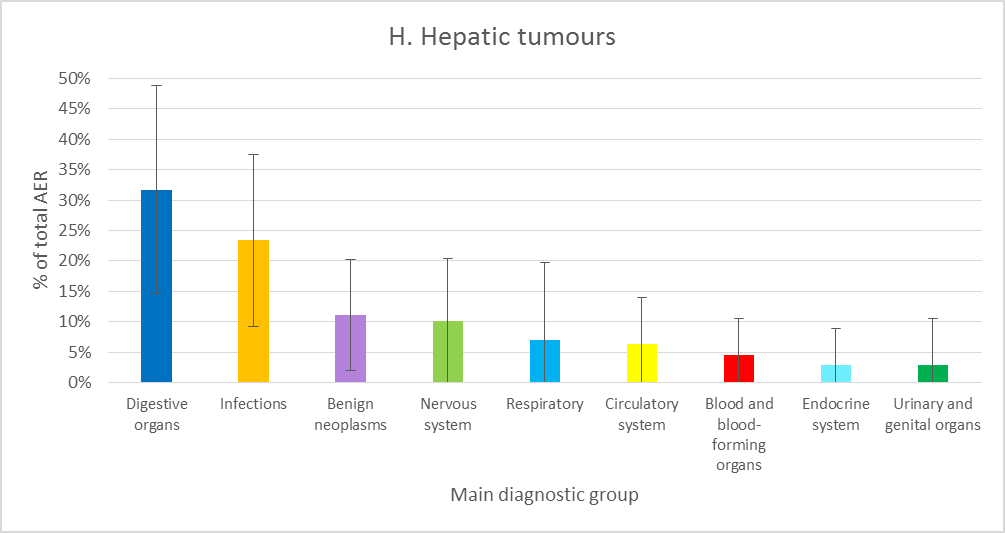
**

**Note. The scale of this figure goes up to 50%. The following main diagnostic groups are not included: Diseases of bone, joint and soft tissue, Diseases of skin and subcutaneous tissue, and New primary cancers were not included in this chart as the AERs for these main diagnostic groups were below 0.**

**
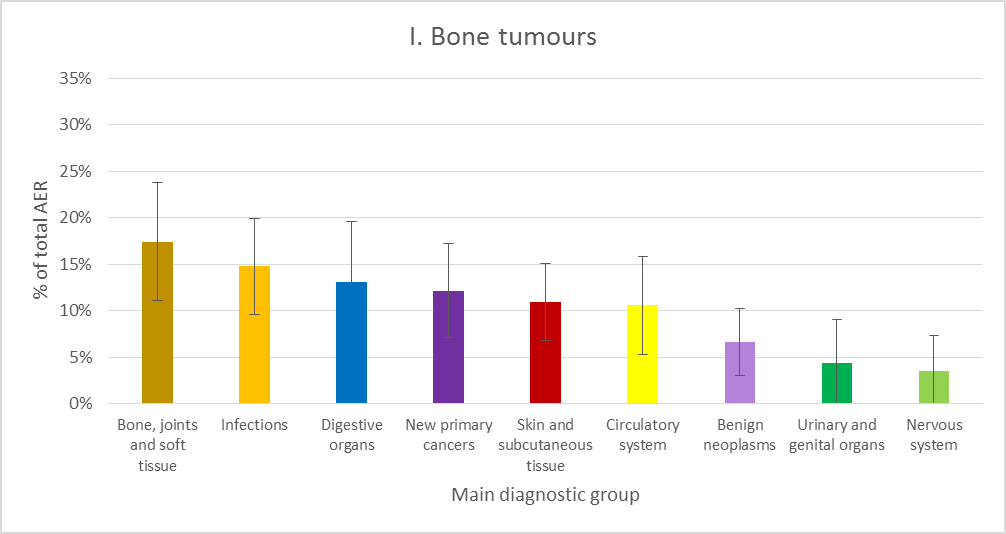
**

**Note. The following main diagnostic groups are not included: Diseases of blood and blood-forming organs (3%), Diseases of the endocrine system (2%), and Diseases of the respiratory system (1%)**

**
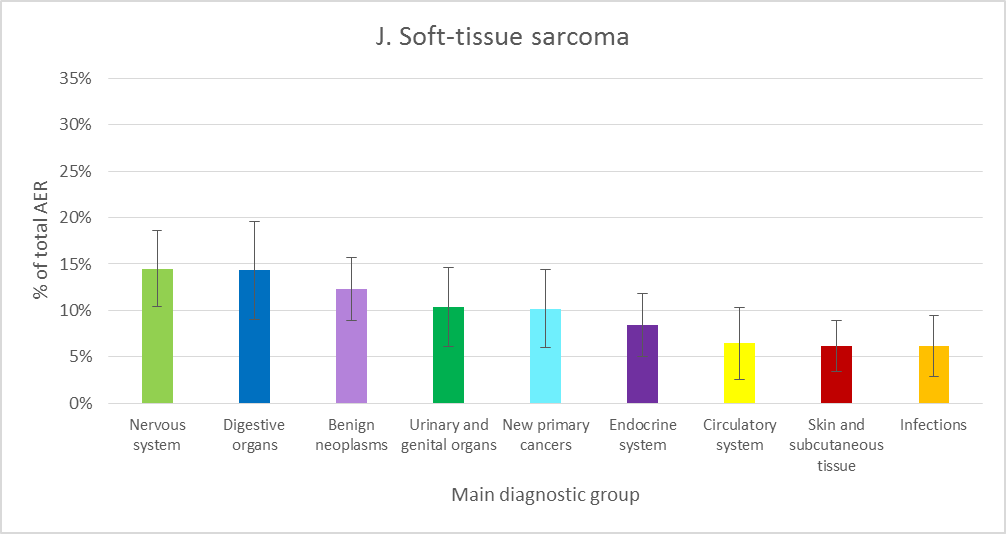
**

**Note. The following main diagnostic groups are not included: Diseases of bone, joints and soft tissue (5%), Diseases of the respiratory system (5%), and Diseases of blood and blood-forming organs (1%)**

**
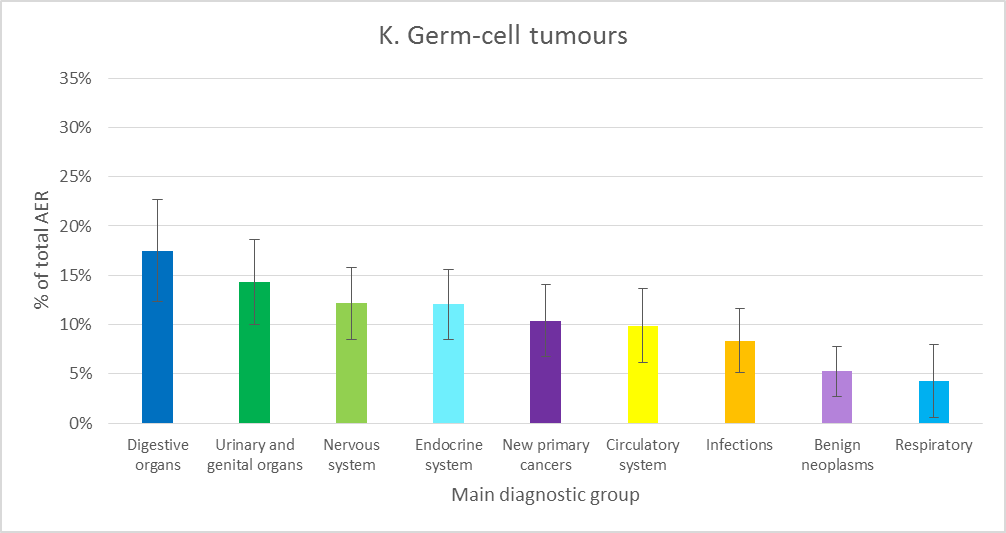
**

**Note. The following main diagnostic groups are not included: Diseases of skin and subcutaneous tissue (4%), Diseases of blood and blood-forming organs (2%), and Diseases of bone, joints and soft tissue (0%)**

**
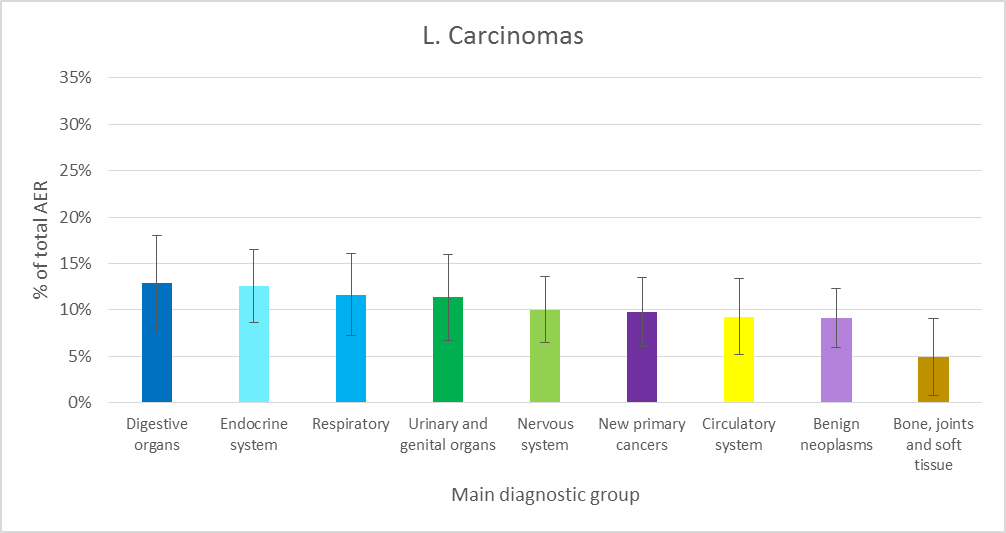
**

**Note. The following main diagnostic groups are not included: Infectious and parasitic diseases (4%), Diseases of skin and subcutaneous tissue (4%), and Diseases of blood and blood-forming organs (0%)**

**
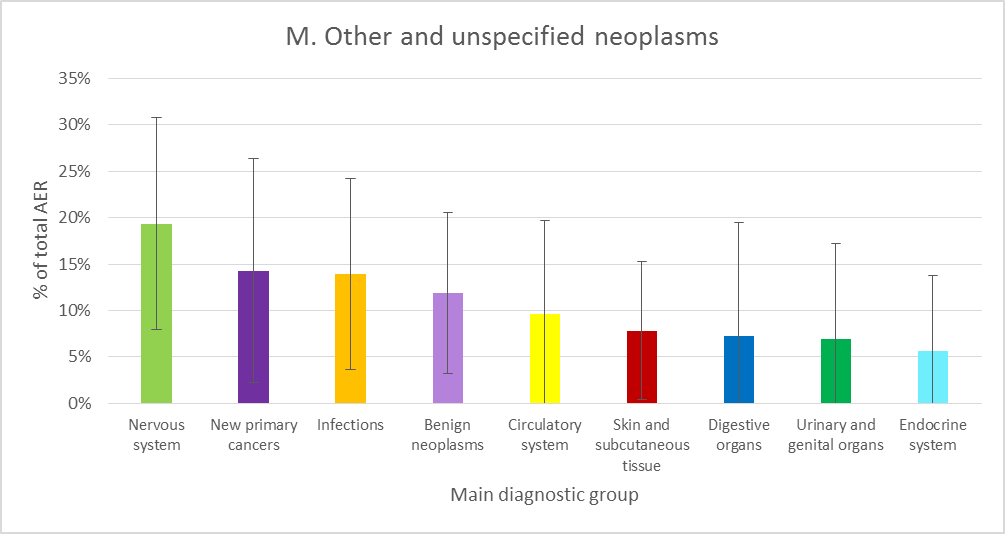
**

**Note. The following main diagnostic groups are not included: Diseases of blood and blood-forming organs (3%) and Diseases of bone, joints and soft tissue (0%). Note that Diseases of the respiratory system were not included in this chart, as the AER for this main diagnostic group was below 0.**
