# Supplementary material for: Long-term inpatient disease burden in the Adult Life after Childhood Cancer in Scandinavia (ALiCCS) study: A cohort study of 21,297 childhood cancer survivors
Source: PLoS Med. 2017 May 9;14(5):e1002296. doi: 10.1371/journal.pmed.1002296 (PMC5423554; doi:10.1371/journal.pmed.1002296)
Supplement: S2 Fig — (A) Leukaemia; (B) Hodgkin lymphoma; (C) non-Hodgkin lymphoma; (D) central nervous system (CNS) tumours; (E) neuroblastoma; (F) retinoblastoma; (G) renal tumours; (H) hepatic tumours; (I) bone tumours; (J) soft-tissue sarcoma; (K) germ-cell tumours, (L) carcinomas; and (M) other and unspecified tumours. Note: other lymphomas are not presented separately, as it is a very small group (n = 205). SBDRs for recurrence of childhood cancer and for new primary cancers by type of childhood cancer are presented in S3 Fig. (DOCX) [file pmed.1002296.s004.docx]

**S2 Figure. Observed (Obs) number of bed days at hospital and associated standardised bed day ratios (SBDRs) for diseases in any of 120 disease categories and for somatic diseases in each of 11 main diagnostic groups by type of childhood cancer.**

(A) Leukaemia; (B) Hodgkin lymphoma; (C) Non-Hodgkin lymphoma; (D) CNS tumours; (E) Neuroblastoma; (F) Retinoblastoma; (G) Renal tumours; (H) Hepatic tumours; (I) Bone tumours; (J) Soft-tissue sarcoma; (K) Germ-cell tumours, (L) Carcinomas; (M) Other and unspecified tumours.

Note: Other lymphomas are not presented separately as it is a very small group (n=205). SBDRs for recurrence of childhood cancer and for new primary cancers by type of childhood cancer are presented in S3 Figure.

**A. Leukaemia**

**B. Hodgkin lymphoma**

**C. Non-Hodgkin lymphoma**

**D. CNS tumours**

Note that Diseases of the nervous system and sense organs have a RR beyond the scale.

**E. Neuroblastoma**

**F. Retinoblastoma**

**G. Renal tumours**

**H. Hepatic tumours**

Note that the RRs for Benign neoplasms and Diseases of blood and blood-forming organs were beyond the scale.

**I. Bone tumours**

**J. Soft-tissue sarcomas**

**K. Germ-cell neoplasms**

**L. Carcinomas**

**M. Other and unspecified neoplasms**

Note that the RR for Diseases of blood and blood-forming organs was beyond the scale.
