# Supplementary material for: Long-term inpatient disease burden in the Adult Life after Childhood Cancer in Scandinavia (ALiCCS) study: A cohort study of 21,297 childhood cancer survivors
Source: PLoS Med. 2017 May 9;14(5):e1002296. doi: 10.1371/journal.pmed.1002296 (PMC5423554; doi:10.1371/journal.pmed.1002296)
Supplement: S3 Fig — (DOCX) [file pmed.1002296.s005.docx]

**S3 Figure. Standardised bed day ratio (SBDR) with cancer recurrence or new primary cancers by childhood cancer type.**
