# Supplementary material for: Long-term inpatient disease burden in the Adult Life after Childhood Cancer in Scandinavia (ALiCCS) study: A cohort study of 21,297 childhood cancer survivors
Source: PLoS Med. 2017 May 9;14(5):e1002296. doi: 10.1371/journal.pmed.1002296 (PMC5423554; doi:10.1371/journal.pmed.1002296)
Supplement: S1 STROBE Checklist — (DOCX) [file pmed.1002296.s006.docx]

STROBE Statement—checklist of items that should be included in reports of observational studies

|  | Item No | Recommendation |
| --- | --- | --- |
| **Title and abstract** | 1 | (*a*) Indicate the study’s design with a commonly used term in the title or the abstract  The study design is stated in the Title.  ***Long-term inpatient disease burden in the Adult Life after Childhood Cancer in Scandinavia (ALiCCS) study: a cohort study of 21 297 childhood cancer survivors*** |
|  |  | (*b*) Provide in the abstract an informative and balanced summary of what was done and what was found  The Abstract in the main manuscript provides a balanced summary of what was done and what was found.  *“****Abstract***  ***Background:*** *Survivors of childhood cancer are at increased risk for a wide range of late effects. However, no large population-based studies have included the whole range of somatic diagnoses including subgroup diagnoses and all main types of childhood cancers. Therefore, we aimed to provide the most detailed overview of the long-term risk of hospitalisation in survivors of childhood cancer.*  ***Methods and findings:*** *From the national cancer registers of Denmark, Finland, Iceland, and Sweden, we identified 21 297 five-year survivors of childhood cancer diagnosed with cancer before the age of 20 years in the period 1943–2008 in Denmark, 1971–2008 in Finland, 1955–2008 in Iceland, and 1958–2008 in Sweden. We randomly selected 152 231 population comparison subjects matched by age, sex, year, and country (or municipality in Sweden) from the national population registers. Using a cohort design, study subjects were followed in the national hospital registers in Denmark, 1977–2010; Finland, 1975–2012; Iceland, 1999–2008; and Sweden, 1968–2009. Disease-specific hospitalisation rates in survivors and comparison subjects were used to calculate survivors’ standardised hospitalisation rate ratios (RRs), absolute excess risks (AERs), and standardised bed day ratios (SBDRs) based on length of stay in hospital. We adjusted for sex, age, and year by indirect standardisation.*  *During 336 554 person-years of follow-up (mean, 16 years; range 0–42 years) childhood cancer survivors experienced 21 325 first hospitalisations for diseases in one or more of 120 disease categories, when 10 999 were expected, yielding an overall RR of 1·94 (95% CI 1·91–1·97). For each additional year of follow-up, an average of 3 of 100 survivors were hospitalised for a new excess disease beyond background rates. The pattern of excess hospitalisations was dominated by diseases of the nervous and endocrine systems, digestive organs, and respiratory tract. Survivors of all types of childhood cancer were at increased, persistent risk for subsequent hospitalisation, the highest risks being those of survivors of neuroblastoma (RR 2·6 [2·4–2·8], n=876), hepatic tumours (RR 2·5 [2·0–3·1], n=92), and central nervous system tumours (RR 2·4 [2·3–2·5], n=6175). Survivors spent on average five times as many days in hospital as comparison subjects (SBDR 4·96 [4·94–4·98], n=422 218).*  *Our study is likely to underestimate the absolute overall disease burden experienced by survivors as less severe late effects are missed if they are treated sufficiently in the out-patient setting or in the primary health care system.*  ***Conclusion:*** *Childhood cancer survivors were at increased long-term risk for diseases requiring inpatient treatment even decades after their initial cancer. Health care providers who do not work in the area of late effects, especially those in the primary health care, should be aware of this highly challenged group of patients in order to avoid or postpone hospitalisations by prevention, early detection, and appropriate treatments.”* |
| Introduction | | |
| Background/rationale | 2 | Explain the scientific background and rationale for the investigation being reported  In the Introduction, second half of the first paragraph and second paragraph a scientific background and rationale is provided.  *“Because of intense exposure to radiation and highly toxic compounds during treatment, a high proportion of survivors of childhood cancer now face somatic, mental, and cognitive late effects, many of which become clinically apparent even decades after the cancer was cured [3-6]. Only a limited number of studies have investigated the hospitalisation patterns subsequent to treatment for cancer diagnosed during childhood, adolescence and young adulthood [7-12]. They all revealed an overall increased risk for hospitalisations. However, the record linkage studies with diagnostic inpatient information (8,9,11,12) were limited by study size and unable to give reliable risk estimates for rare types of childhood cancer or combinations of types of childhood cancer and types of subsequent disease. The larger studies were either based on self-reported information on causes for hospitalisation (7), or questionnaire data from patients’ primary health care physicians (10) and with relatively large proportions of survivors lost to follow-up.*  *In a population-based cohort study with virtually no loss to follow-up and exclusive use of medically verified diagnostic information from individual inpatient records, we studied the full range of somatic morbid conditions requiring hospitalisation in 21 297 five-year survivors of childhood cancer diagnosed in 1943–2008. As this is the largest long-term follow-up study of inpatient care among childhood cancer survivors conducted so far, it allowed stratifications and detailed analyses that were not possible in previous studies and provides novel information on diseases that first become symptomatic in middle age or senescence.”* |
| Objectives | 3 | State specific objectives, including any prespecified hypotheses  The objectives are stated in the last sentence of the introduction.  *“The primary aim of our study was to present a comprehensive and yet detailed overview of the long-term frequency and distribution of somatic diseases serious enough to require hospitalisations in five-year survivors of childhood cancer combined and by type of cancer.”* |
| Methods | | |
| Study design | 4 | Present key elements of study design early in the paper  The key elements of our study design are presented in the first line of the methods section and in the following paragraph.  *“This retrospective, register-based cohort study is part of the collaborative study Adult Life after Childhood Cancer in Scandinavia (ALiCCS)[13] (*[*www.aliccs.org*](http://www.aliccs.org)*).*  *The basic childhood cancer cohort in the present analysis is a sub-cohort of the Nordic ALiCCS material, comprising 33 576 individuals with cancer diagnosed in Denmark, Finland, Iceland or Sweden in people under the age of 20 years, between the start of the cancer registries (Denmark 1943; Finland 1953; Iceland 1955 and Sweden 1958) and 31 December 2008 (Figure 1) [14-17]. Patients from Norway were not included as complete hospitalisation histories with all diseases included in the present study were not available. From the cancer registries, we obtained each patient’s personal identification number, date of diagnosis and type of cancer and assigned patients to the 12 main diagnostic groups of the International Classification Scheme for Childhood Cancer, with lymphoma divided further into Hodgkin and non-Hodgkin lymphoma [18]. For each childhood cancer patient, we selected five comparison subjects from the national population registers, who were alive on the date of cancer diagnosis of the corresponding patient, of the same sex, age, and country, and without a cancer diagnosis before the age of 20 years.”* |
| Setting | 5 | Describe the setting, locations, and relevant dates, including periods of recruitment, exposure, follow-up, and data collection  The included countries and recruitment periods are describe in the second paragraph in the methods section.  *“The basic childhood cancer cohort in the present analysis is a sub-cohort of the Nordic ALiCCS material, comprising 33 576 individuals with cancer diagnosed in Denmark, Finland, Iceland or Sweden in people under the age of 20 years, between the start of the cancer registries (Denmark 1943; Finland 1953; Iceland 1955 and Sweden 1958) and 31 December 2008.”*  The follow-up periods are described in the statistical analysis of the methods section.  *“Follow-up for diseases but cancer was started five years after the date of cancer diagnosis for the cancer survivors and the corresponding date for the comparison subjects or at the beginning of the hospital registers (Denmark, 1977; Finland, 1975; Iceland, 1999; Sweden, from 1968–1987 stepwise inclusion of counties and nationwide since 1987), whichever occurred later. Follow-up for a second cancer in survivors and a first cancer in comparisons started at age 20 years at the earliest. Follow-up ended on date of death, date of emigration, or end of study (Iceland: 31 December 2008; Sweden: 31 December 2009; Denmark: 31 October 2010; Finland: 31 December 2012), whichever occurred first.”* |
| Participants | 6 | (*a*) *Cohort study*—Give the eligibility criteria, and the sources and methods of selection of participants. Describe methods of follow-up  Survivors included in the study had been diagnosed with cancer before the age of 20 years in Denmark, Finland, Iceland or Sweden. The participants are described in the second paragraph in the methods section.  *“The basic childhood cancer cohort in the present analysis is a sub-cohort of the Nordic ALiCCS material, comprising 33 576 individuals with cancer diagnosed in Denmark, Finland, Iceland or Sweden in people under the age of 20 years, between the start of the cancer registries (Denmark 1943; Finland 1953; Iceland 1955 and Sweden 1958) and 31 December 2008.”*    Exclusions are described in Figure 1 (flowchart) and in the third paragraph in the methods section and in the second paragraph under Hospital admissions:  *“Before linkage of study subjects to the respective national hospital registers, we excluded those in whom more than one cancer was diagnosed during childhood; those who had died or emigrated before the start of the national hospital registers (Sweden, stepwise inclusion of counties in 1964–1987 and nationwide since 1987; Finland, 1975; Denmark, 1977; Iceland, 1999); and those who had died or emigrated within five years of the date of cancer diagnosis or an equivalent time lag for the population comparisons.“*  *“We excluded cancer survivors and comparison subjects who had ever been hospitalised with a congenital malformation or chromosome abnormality (ICD-8 codes 740−759, ICD-10 codes Q00−Q99), leaving 21 297 five-year survivors of childhood cancer and 152 231 population comparison subjects for the risk analysis.”*  Methods of follow-up is described in the second paragraph under Hospital admissions and first paragraph under Statistical analysis.  *“Data on cancer survivors and comparison subjects were linked to the hospital registers, and a full hospital history with discharge diagnoses was established for each person with a previous hospital contact.”*  *“Follow-up for diseases but cancer was started five years after the date of cancer diagnosis for the cancer survivors and the corresponding date for the comparison subjects or at the beginning of the hospital registers (Denmark, 1977; Finland, 1975; Iceland, 1999; Sweden, from 1968–1987 stepwise inclusion of counties and nationwide since 1987), whichever occurred later. Follow-up for a second cancer in survivors and a first cancer in comparisons started at age 20 years at the earliest. Follow-up ended on date of death, date of emigration, or end of study (Iceland: 31 December 2008; Sweden: 31 December 2009; Denmark: 31 October 2010; Finland: 31 December 2012), whichever occurred first.”*  *Case-control study*—Give the eligibility criteria, and the sources and methods of case ascertainment and control selection. Give the rationale for the choice of cases and controls. Not relevant.  *Cross-sectional study*—Give the eligibility criteria, and the sources and methods of selection of participants. Not relevant. |
|  |  | (*b*) *Cohort study*—For matched studies, give matching criteria and number of exposed and unexposed  The matching criteria are described in the second paragraph of the methods section.  “For each childhood cancer patient, we selected five comparison subjects from the national population registers, who were alive on the date of cancer diagnosis of the corresponding patient, of the same sex, age, and country, and without a cancer diagnosis before the age of 20 years. Fewer than five comparison subjects were available for 157 patients”  The final number of survivors (exposed) and comparisons (unexposed) to be included for analyses are presented in the last sentence of the second paragraph under Hospital admissions. This is also presented in a flow chart (Figure 1).  “…leaving 21 297 five-year survivors of childhood cancer and 152 231 population comparison subjects for the risk analysis.”  *Case-control study*—For matched studies, give matching criteria and the number of controls per case. Not relevant. |
| Variables | 7 | Clearly define all outcomes, exposures, predictors, potential confounders, and effect modifiers. Give diagnostic criteria, if applicable  Definition of exposure:  *“…assigned patients to the 12 main diagnostic groups of the International Classification Scheme for Childhood Cancer, with lymphoma divided further into Hodgkin and non-Hodgkin lymphoma [18].”*  Furthermore, childhood cancer was defined as cancer before the age of 20 years:  *“…with cancer diagnosed in Denmark, Finland, Iceland or Sweden in people under the age of 20 years…”*  Definition of outcomes:  *“To quantify the inpatient disease burden among study subjects comprehensibly, we grouped the hospital discharge diagnoses into 120 disease categories or diagnoses, which in turn were assembled into 12 main diagnostic groups (Supplementary Table 1). Diagnoses coded according to ICD-7, ICD-9, and ICD-10 were adapted to ICD-8 to the extent possible, as shown in the table.“*  Potential confounders:  *“We excluded cancer survivors and comparison subjects who had ever been hospitalised with a congenital malformation or chromosome abnormality (ICD-8 codes 740−759, ICD-10 codes Q00−Q99) as this could possibly confound the associations between childhood cancer and several of the outcomes,…”* |
| Data sources/ measurement | 8* | For each variable of interest, give sources of data and details of methods of assessment (measurement). Describe comparability of assessment methods if there is more than one group.  Information on childhood cancer is described in the middle of the second paragraph of the methods section.  *“From the cancer registries, we obtained each patient’s personal identification number, date of diagnosis and type of cancer and assigned patients to the 12 main diagnostic groups of the International Classification Scheme for Childhood Cancer, with lymphoma divided further into Hodgkin and non-Hodgkin lymphoma.”*  The hospital registers and outcome variables are described in the first and second paragraph under Hospital admissions  *“The nationwide hospital registries hold information on all non-psychiatric hospital admissions in the four countries [19, 20]. Registration is mandatory, and the treating physician submits diagnostic information electronically. Each admission to hospital initiates a record, which includes the personal identification number of the patient, dates of admission and discharge, a primary discharge diagnosis, and supplementary diagnoses coded according to the International Classification of Diseases 7th–10th revisions (ICD-7–ICD-10).*  *Data on cancer survivors and comparison subjects were linked to the hospital registers, and a full hospital history with discharge diagnoses was established for each person with a previous hospital contact.”*  The outcome variables are described in the third paragraph under hospital admissions.  *“To quantify the inpatient disease burden among study subjects comprehensibly, we grouped the hospital discharge diagnoses into 120 disease categories or diagnoses, which in turn were assembled into 12 main diagnostic groups (Supplementary Table 1). Diagnoses coded according to ICD-7, ICD-9, and ICD-10 were adapted to ICD-8 to the extent possible, as shown in the table.”* |
| Bias | 9 | Describe any efforts to address potential sources of bias  To eliminate the risk of loss-to follow-up, all death and emigrations were identified in the national population registers as described in the methods section,last sentence in the second paragraph.  *“For both patients and comparison subjects, we obtained information from the population registers on vital status and emigration during follow-up.”*  To exclude the risk of recall bias, we used register-based information on hospitalisations from high quality hospital registers. This is described under Hospital admissions in the methods section  *“The nationwide hospital registries hold information on all non-psychiatric hospital admissions in the four countries [19, 20]. Registration is mandatory, and the treating physician submits diagnostic information electronically. Each admission to hospital initiates a record, which includes the personal identification number of the patient, dates of admission and discharge, a primary discharge diagnosis, and supplementary diagnoses coded according to the International Classification of Diseases 7th–10th revisions (ICD-7–ICD-10)”*  To eliminate the risk of selection bias in the comparison cohort, they were randomly selected from population-based and nationwide population registers. This was described in the second paragraph of the methods section.  *“For each childhood cancer patient, we randomly selected five comparison subjects from the national population registers, who were alive on the date of cancer diagnosis of the corresponding patient, of the same sex, age, and country, and without a cancer diagnosis before the age of 20 years.”* |
| Study size | 10 | Explain how the study size was arrived at  Please see our flow chart in Figure 1 in addition with the inserted text below from the methods section.  *“The basic childhood cancer cohort in the present analysis is a sub-cohort of the Nordic ALiCCS material, comprising 33 576 individuals with cancer diagnosed in Denmark, Finland, Iceland or Sweden in people under the age of 20 years, between the start of the cancer registries (Denmark 1943; Finland 1953; Iceland 1955 and Sweden 1958) and 31 December 2008 (Figure 1) [14-17].”*  *“For each childhood cancer patient, we selected five comparison subjects from the national population registers, who were alive on the date of cancer diagnosis of the corresponding patient, of the same sex, age, and country, and without a cancer diagnosis before the age of 20 years. Fewer than five comparison subjects were available for 157 patients, leaving 167 712 individuals for study. For both patients and comparison subjects, we obtained information from the population registers on vital status and emigration during follow-up.*  *Before linkage of study subjects to the respective national hospital registers, we excluded those in whom more than one cancer was diagnosed during childhood; those who had died or emigrated before the start of the national hospital registers (Sweden, stepwise inclusion of counties in 1964–1987 and nationwide since 1987; Finland, 1975; Denmark, 1977; Iceland, 1999); and those who had died or emigrated within five years of the date of cancer diagnosis or an equivalent time lag for the population comparisons. These exclusions resulted in cohorts of 21 518 five-year childhood cancer survivors and 152 481 population comparison subjects (Figure 1).”*  *“We excluded cancer survivors and comparison subjects who had ever been hospitalised with a congenital malformation or chromosome abnormality (ICD-8 codes 740−759, ICD-10 codes Q00−Q99) as this could possibly confound the associations between childhood cancer and several of the outcomes, leaving 21 297 five-year survivors of childhood cancer and 152 231 population comparison subjects for the risk analysis (Figure 1).”* |
| Quantitative variables | 11 | Explain how quantitative variables were handled in the analyses. If applicable, describe which groupings were chosen and why  Not relevant, as we only included binary outcome variables. |
| Statistical methods | 12 | *(*a) Describe all statistical methods, including those used to control for confounding  The statistical methods are described under the heading “Statistical analysis” in the end of the methods section after a description of the follow-up periods.  *“Only the primary diagnosis, i.e. the main reason for hospitalisation at each in-patient contact, was included in the analyses. If participants had more than one hospital admission for a particular disease category, only the first record was retained. Risk was analysed for each of the 120 disease categories, and the numbers of first hospitalisations for somatic diseases in different categories were added up for the 12 main diagnostic groups. For each person, the final sum yielded the total number of first hospitalisations for diseases requiring hospitalisation in different categories. The observed number of first hospital admissions of survivors of childhood cancer for a given disease category were compared with expected numbers derived from the appropriate sex-, age- and calendar period-specific hospitalisation rates of the comparison cohort, and the standardised hospitalisation rate ratios (RRs) were estimated. The significance and 95% CIs were computed using Fieller’s theorem and assuming that the observed number of first hospital contacts follows a Poisson distribution [21]. The absolute excess risk (AER), i.e. the additional risk for hospitalisation above background levels, was derived as the difference between the observed and expected first hospitalisation rates for a particular disease category per 100 000 person-years of follow-up.*  *Using the same methods as for the RR, we also added up the total number of bed days spent in hospital for cancer survivors and the number expected had they had the sex-, age- and calendar period-specific bed day rates of the comparison subjects. We thus derived standardised bed day ratios (SBDRs) for cancer survivors. In the analyses of bed days, we included not only the first hospitalisation for a given disease category but all hospitalisations for diseases, cancer recurrences included, of the ICD sections of interest.”* |
|  |  | (*b*) Describe any methods used to examine subgroups and interactions |
|  |  | (*c*) Explain how missing data were addressed |
|  |  | (*d*) *Cohort study*—If applicable, explain how loss to follow-up was addressed  Not relevant, as we had no loss to follow-up due to the personal identification numbers of all residents in the Nordic countries and existence of the population registers.  “For both patients and comparison subjects, we obtained information from the population registers on vital status and emigration during follow-up.”  *Case-control study*—If applicable, explain how matching of cases and controls was addressed  *Cross-sectional study*—If applicable, describe analytical methods taking account of sampling strategy |
|  |  | (*e*) Describe any sensitivity analyses |

Continued on next page

| Results | | |
| --- | --- | --- |
| Participants | 13* | (a) Report numbers of individuals at each stage of study—eg numbers potentially eligible, examined for eligibility, confirmed eligible, included in the study, completing follow-up, and analysed  We have presented this in detail in a flow chart (Figure 1) and it is described in the Methods section (also see item no. 10) |
|  |  | (b) Give reasons for non-participation at each stage |
|  |  | (c) Consider use of a flow diagram See Figure 1 |
| Descriptive data | 14* | (a) Give characteristics of study participants (eg demographic, clinical, social) and information on exposures and potential confounders  See Table 1 for main characteristics of study participants. |
|  |  | (b) Indicate number of participants with missing data for each variable of interest |
|  |  | (c) *Cohort study*—Summarise follow-up time (eg, average and total amount)  The follow-up time is presented in the second line of the results section.  *“The survivors were monitored in the national hospital registers for 336 554 person-years (mean, 16 years; range, 0–42 years).”* |
| Outcome data | 15* | *Cohort study*—Report numbers of outcome events or summary measures over time  In the second paragraph of the results section overall numbers of hospitalisations is presented.  *“Overall, 9698 (45·5%) childhood cancer survivors were ever admitted to hospital for somatic disease, when 5399·2 (25·4%) were expected, yielding a RR of 1·80 (1·76–1·84). The 9698 survivors ever hospitalised experienced 21 325 first admissions to hospital for diseases in one or more of the 120 disease categories listed in Supplementary Table 1, when 10 999·0 were expected, yielding an overall RR for a new category-specific admission of 1·94, Table 2.”* |
|  |  | *Case-control study—*Report numbers in each exposure category, or summary measures of exposure Not relevant |
|  |  | *Cross-sectional study—*Report numbers of outcome events or summary measures Not relevant |
| Main results | 16 | (*a*) Give unadjusted estimates and, if applicable, confounder-adjusted estimates and their precision (eg, 95% confidence interval). Make clear which confounders were adjusted for and why they were included |
|  |  | (*b*) Report category boundaries when continuous variables were categorized Not relevant |
|  |  | (*c*) If relevant, consider translating estimates of relative risk into absolute risk for a meaningful time period  “Based on the observed and expected hospitalisation rates of 6336·3 and 3268·1 per 100 000 person-years, respectively, the AER of survivors for a new category-specific admission to hospital was 3068 per 100 000 person-years (Table 2). Thus, for each additional year of follow-up, approximately three of 100 survivors of childhood cancer were hospitalised for a new excess disease.” |
| Other analyses | 17 | Report other analyses done—eg analyses of subgroups and interactions, and sensitivity analyses  We stratified results by sex, cancer type, attained age and main diagnostic groups of somatic disease.  These results are presented in Table 2-4 and Figure 2, 3A, 3B, 4 and 5. |
| Discussion | | |
| Key results | 18 | Summarise key results with reference to study objectives  The key results are stated in the first paragraph of the discussion.  *“****Discussion***  *This population-based study of 21 297 five-year survivors of childhood cancer in the Nordic countries gives an extensive overview of the pattern of later somatic conditions that are serious enough to require inpatient care. The study shows that survivors are hospitalised because of a new somatic disease twice as often as population comparisons and that they spend five times as many days in hospital. Although cancer and its treatment may affect practically all organ systems adversely, the pattern of diseases requiring hospitalisation of survivors varied widely by type of childhood cancer and by survivors’ attained age. Despite the variations, however, the important findings are that the majority of childhood cancer survivors are at substantial risk for late effects requiring inpatient care and that the risk remains increased throughout life.”* |
| Limitations | 19 | Discuss limitations of the study, taking into account sources of potential bias or imprecision. Discuss both direction and magnitude of any potential bias  The limitations and implications are discussed in the fourth and fifth paragraph of the discussion.  *“We used hospital-based diagnoses made by physicians as markers of disease outcome. Although this approach increased the validity of the diagnostic information, less severe late effects might have been missed because they were treated sufficiently as hospital out-patients or in the primary health care system. This implies that we almost certainly underestimate the absolute overall somatic disease burden experienced by childhood cancer survivors. As this limitation also applies to the comparison cohort, however, the validity of the relative risk estimates is acceptable, although restricted to conditions that require a hospital contact. Moreover, we cannot exclude the possibility that our results were affected by better medical surveillance of the survivors than the population comparisons, which could explain part of the longer hospital stays in the survivors. Not covered by the present study of the somatic disease burden, but important to stress, is the fact that many childhood cancer survivors face additional and sometime significant challenges due to cognitive and other psychological adverse effects from cancer and its treatment (3).*  *The information on treatment currently included in the Nordic cancer registries is generally too crude or absent for meaningful analyses of type and dose of chemotherapy and radiation and specific disease outcomes. Although our study does not attribute causality, this comprehensive overview provides important clinical information on the lifelong inpatient disease burden experienced by childhood cancer survivors overall and of a number of patient characteristics, including type of childhood cancer, type of late effect, sex, and attained age. Associations, including dose-response effects, between specific treatment regimens and the risk of selected late effects are being addressed in clinical case-cohort studies within the ALiCCS cohort [13].”* |
| Interpretation | 20 | Give a cautious overall interpretation of results considering objectives, limitations, multiplicity of analyses, results from similar studies, and other relevant evidence  The interpretation is discussed in the last paragraph of the discussion.  *“In conclusion, we found that survivors of childhood cancer have a highly increased long-term disease burden, with a broad range of late effects that require inpatient treatment and substantially longer stays in hospital as compared with the background population of similar age and sex. This will inevitably constitute a growing health care challenge for our society, affecting medical costs, and may profoundly influence the quality of life and life expectancy of childhood cancer survivors. Our findings underscore the need for continued follow-up of survivors, with particular focus on survivors of neuroblastoma, hepatic tumours, CNS tumours, Hodgkin lymphoma and leukaemia. In particular, primary health care physicians should be aware of the risk for second primary cancers in patients who are childhood cancer survivors, as the relative risks for cancers are high and tumours may appear earlier in life than usual.”* |
| Generalisability | 21 | Discuss the generalisability (external validity) of the study results  The generalizability is discussed in the sixth paragraph of the discussion.  *“As our study is population-based and include a randomly selected comparison group and data from high quality health registers, we consider our results valid for children treated for cancer in other countries with similar health care systems.”* |
| Other information | | |
| Funding | 22 | Give the source of funding and the role of the funders for the present study and, if applicable, for the original study on which the present article is based  As per PLOS Medicines guidance, we have not included funding sources in the Acknowledgements or anywhere else in the manuscript. Funding information has been entered in the financial disclosure section of the online submission system. As follows:  *“This study was funded by grant no. 09-066899 from the Danish Council for Strategic Research”.* |

*Give information separately for cases and controls in case-control studies and, if applicable, for exposed and unexposed groups in cohort and cross-sectional studies.

**Note:** An Explanation and Elaboration article discusses each checklist item and gives methodological background and published examples of transparent reporting. The STROBE checklist is best used in conjunction with this article (freely available on the Web sites of PLoS Medicine at http://www.plosmedicine.org/, Annals of Internal Medicine at http://www.annals.org/, and Epidemiology at http://www.epidem.com/). Information on the STROBE Initiative is available at www.strobe-statement.org.
